# Evaluating the impact of cell-penetrating motif position on the cellular uptake of magnetite nanoparticles

**DOI:** 10.3389/fbioe.2024.1450694

**Published:** 2024-12-02

**Authors:** Laura Salgado, Paula C. Cifuentes-Delgado, Juan Camilo Orozco, Carolina Muñoz-Camargo, Luis H. Reyes, Valentina Quezada, Juan C. Cruz

**Affiliations:** ^1^ Department of Biomedical Engineering, Universidad de Los Andes, Bogotá, Colombia; ^2^ Center for Microscopy (MicroCore), Vice Presidency for Research and Creation, Universidad de Los Andes, Bogotá, Colombia; ^3^ Product and Process Design Group (GDPP), Department of Chemical and Food Engineering, Universidad de Los Andes, Bogotá, Colombia

**Keywords:** cell-penetrating peptides, energy-dependent cellular uptake, clathrin-mediated endocytosis, magnetite nanoparticles, cell-penetrating motif

## Abstract

Cell-penetrating peptides (CPPs) have been employed to enhance the cellular uptake and intracellular delivery of various nanocarriers. Among them, nanoparticles (NPs) have been used as suitable vehicles for delivering different bioactive molecules in the treatment of a diverse range of diseases. Given the pivotal role of the conjugation method of CPPs, this study aims to evaluate the impact of the position of a cell-penetrating motif (LFVCR) on the biocompatibility, cellular uptake, and endosomal escape of magnetite NPs. The designed peptide’s physicochemical properties suggest they are well-suited for efficient cell penetration with minimal cytotoxicity. The resulting designed nanoconjugates were characterized using Fourier transform infrared spectroscopy (FTIR), thermogravimetric analysis (TGA), dynamic light scattering (DLS), and transmission electron microscopy (TEM). The results indicate that motif position significantly impacts the cellular uptake and endosomal escape of the designed nanobioconjugates. Key findings suggest that motif exposure enhances endocytosis-mediated cell internalization and improves endosomal escape efficiency. These results were compared with nanobioconjugates displaying previously reported CPPs. The selected nanobioconjugate demonstrated superior performance in endosomal escape and comparable cell uptake to the reference nanobioconjugates. These results, along with the nanobioconjugate’s physicochemical characteristics and high biocompatibility, position the nanocarrier as a suitable candidate for delivering diverse bioactive molecules.

## Introduction

Cell-penetrating peptides (CPPs) are renowned for their ability to penetrate cell membranes at low micromolar concentrations without causing significant membrane damage. Typically, CPPs are short, water-soluble peptides that are partially hydrophobic and/or polybasic, comprising no more than 35 amino acid residues and exhibiting a net positive charge at physiological pH (Madani et al., 2011). Since the discovery in 1988 of the first CPPs, derived from the HIV-1 encoded TAT protein, specifically TAT (48–60) (Green and Loewenstein, 1988), numerous CPPs have been studied, including penetratin, R9, TP10, pVEC, Pep-1, LL-37, VP22, and pISL (Lundberg and Langel, 2003; El-Andaloussi et al., 2005; Järver and Langel, 2006). CPPs often contain one or more motifs—small amino acid sequence patterns—that are associated with their cell-penetrating capacity. A well-known motif is the arginine–glycine–aspartate (RGD) motif, which facilitates translocation into target cells through cell membrane integrin receptors (Hynes, 2002). CPPs have been demonstrated to deliver a wide range of bioactive molecules, including proteins, peptides, oligonucleotides, and nanocarriers, to various cell types and cellular compartments, both *in vivo* and *in vitro* (Järver and Langel, 2006; Koren and Torchilin, 2012). The two primary cellular uptake mechanisms for CPPs include non-endocytotic (energy-independent) pathways and endocytotic pathways. The choice of uptake mechanism depends on the specific characteristics of the CPP, the cargo molecule, the cell type, and the membrane lipid composition (Trabulo et al., 2010; Madani et al., 2011).

The first step of energy-independent mechanisms involves the interaction of positively charged CPPs with negatively charged components of the cell membrane, as well as with the phospholipid bilayer. Most cationic CPPs contain arginine in their sequences. This amino acid has a guanidine head group that can form hydrogen bonds with negatively charged phosphates and sulfates on the cell membrane, facilitating internalization at physiological pH (Farkhani et al., 2014). This cationic amino acid mediates the interaction of the peptide with anionic/acidic motifs on the cell membrane in a receptor-independent manner (Bolhassani, 2011; Koren and Torchilin, 2012). Mechanisms proposed for this internalization include inverted micelle formation (Derossi et al., 1996), pore formation (Matsuzaki et al., 1996), the carpet-like model (Pouny et al., 1992), and the membrane thinning model (Lee et al., 2005). These mechanisms are highly dependent on peptide concentration, sequence, and the lipid composition of the membrane in each study (Madani et al., 2011). On the other hand, energy-dependent mechanisms for CPP uptake involve interactions with extracellular heparan sulfate (Console et al., 2003) and different types of endocytosis (Fittipaldi et al., 2003; Richard et al., 2003), such as macropinocytosis, clathrin-dependent endocytosis, caveolae-dependent endocytosis, and clathrin- and caveolae-independent endocytosis. This diversity suggests that CPP membrane translocation can occur through multiple pathways simultaneously, or that different peptides utilize different uptake mechanisms depending on their cargo and biophysical properties (Fischer et al., 2002). The uptake mechanism can also be influenced by the nature of the cargo, whether the peptides form a stable complex with their cargo, whether the cargo is covalently bound to a CPP, or how the cargo is attached (Silhol et al., 2002).

Nanoparticles (NPs) have been extensively used as therapeutic agents for cancer treatment. However, related research has also explored nanoparticle-mediated therapy for infectious, autoimmune, cardiovascular, neurodegenerative, ocular, and pulmonary diseases (Yetisgin et al., 2020). In drug delivery, NPs have emerged as suitable carriers to improve the bioavailability of hydrophobic drugs, reduce drug degradation, enable sustained and triggered release, offer targeted tumor delivery, and bind or encapsulate multiple drug molecules. They also address solubility and stability issues, thereby prolonging the circulation half-life of the drug (Zhao and Stenzel, 2018; Zeng et al., 2022; Yusuf et al., 2023). CPPs have been employed to enhance the cellular uptake and intracellular delivery of NPs, resulting in a variety of promising NP-CPP vehicles (Rong et al., 2018; Spicer et al., 2018; Wei et al., 2018; Silva et al., 2019). However, the interplay between CPPs and NPs is highly complex, and the choice of the conjugation method plays a pivotal role. It can lead to highly efficient intracellular vectors or, conversely, impair peptide functionality. Covalent linkage of CPPs to NPs is the most prominent modification strategy, providing high stability and precise control over site-selectivity, key requisites for preserving the function and properties of the peptides (Gessner and Neundorf, 2020). Nonetheless, chemical interactions between the functional groups of the peptide and those of the NPs can significantly influence the formation of the secondary structure and the preservation of bioactivity. Our research indicates that CPPs can lose or diminish their cell-penetrating capacity if the motif is not adequately exposed during the immobilization process. However, this issue has not been extensively studied. We believe that understanding this effect is crucial for developing functional nanocarriers that can be effectively translated to clinical applications.

This study aims to preliminarily evaluate the effect of the cell-penetrating motif position on the final biocompatibility, cell internalization, endosomal escape, and activation of clathrin-mediated endocytosis of magnetic NPs. Three peptides were designed using the cell-penetrating motif LFVCR, previously identified by our research group (Ruiz Puentes et al., 2022). The motif was placed at the N-terminal, middle, and C-terminal positions of the final sequences. The sequences were immobilized via the peptide C-terminal on magnetite (Fe_3_O_4_) nanoparticles (MNPs) using a carbodiimide-based coupling strategy. MNPs are widely used in material science, biochemistry, diagnostics, magnetic drug and gene delivery, hyperthermia, magnetic resonance imaging, and theranostics due to their biocompatibility, high saturation magnetization, chemical stability, large surface area, and ease of functionalization (Petrov and Chubarov, 2022). Additionally, we have found that MNPs are suitable nanocarriers for the delivery of drugs, plasmids, siRNA, and other (bio) molecules (Cuellar et al., 2018; Perez et al., 2019; Lopez-Barbosa et al., 2020; Ramírez-Acosta et al., 2020; Cifuentes et al., 2023). Polyethylene glycol (PEG) was employed as a spacer molecule to avoid steric hindrance on the particle surface, and the peptides were attached to PEGylated MNPs. The nanobioconjugates were characterized by their size, surface charge, thermal stability, biocompatibility, cell uptake and endosomal escape capabilities. Cell uptake and endosomal escape properties were compared with those of nanobioconjugates with previously reported CPPs, including the well-studied antimicrobial and cell-penetrating peptide Buforin II (TRSSRAGLQFPVGRVHRLLRK) (Park et al., 1996; Park et al., 2000; Fleming et al., 2008; Cuellar et al., 2018; Sadeghi et al., 2022), as well as two peptides identified by our research group: MS12 (MFVFLVLLPLVS) (Henao et al., 2022) and RD10 (RTLFVCRVGD), the CPP from which the motif was originally discovered (Ruiz Puentes et al., 2022).

## Materials and methods

### Materials

Iron (II) chloride tetrahydrate (98%), iron (III) chloride hexahydrate (97%), acetic acid glacial (99.7%), sodium hydroxide (NaOH) (98%), and tetramethylammonium hydroxide (TMAH) (40%) were obtained from PanReac AppliChem (Barcelona, Spain). (3-aminopropyl) triethoxysilane (APTES) (98%), N-[3-(dimethylamino)-propyl]-N′-ethylcarbodiimide hydrochloride (EDC) (98%), N-hydroxysuccinimide (NHS) (98%), sodium chloride (NaCl) (99%), glutaraldehyde (25%), amine-PEG_12_-amine (NH_2_-PEG_12_-NH_2_), rhodamine B (95%), phosphate buffered saline (PBS, pH 7.2), Thrombin, Triton X-100 (laboratory grade), 3-[4,5-dimethylthiazol-2-yl]-2,5-diphenyltetrazolium bromide (MTT) and Dynasore hydrate were purchased from Sigma-Aldrich (St. Louis, MO, United States). Fetal bovine serum (FBS), and trypsin were obtained from BioWest (Riverside, MO, United States). Dulbecco’s modified Eagle’s medium (DMEM) was purchased from Lonza (Basel, Switzerland). 3-[(2-aminoethyl) dithiol] propionic acid (AEDP) and Hoechst 33342 were purchased from Thermo Fisher Scientific (Waltham, MA, United States). LysoTracker Green DND-26 was purchased from Novus Biologicals (Novus Biologicals, Centennial, CO, United States). Buforin II (TRSSRAGLQFPVGRVHRLLRK), MS12 (MFVFLVLLPLVS), RD10 (RTLFVCRVGD), LG11 (LFVCRSGCFTG), SG11 (SGCLFVCRFTG), and SR11 (SGCFTGLFVCR) were synthesized by GL Biochem Shanghai (Shanghai, China). Biocompatibility assays were conducted in Vero cells (ATCC^®^CCL-81, ATCC, St. Cloud, MN, United States).

### Peptide design and physicochemical properties

**FIGURE 1:**
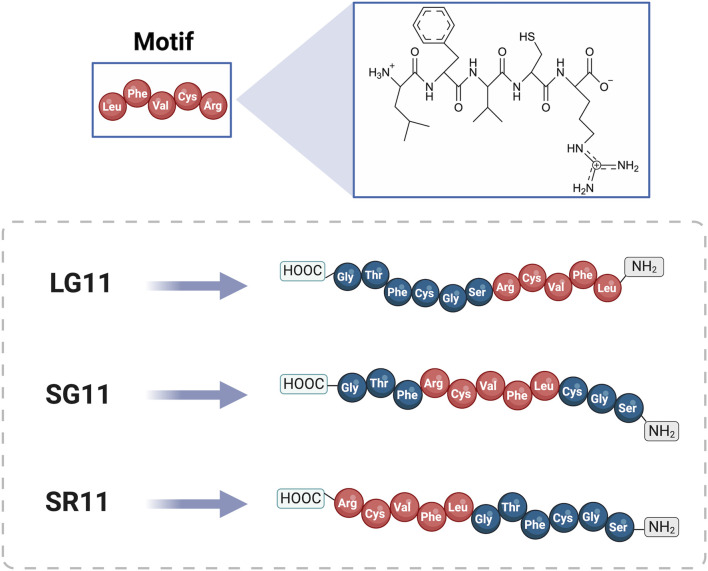
General structure of the designed peptides LG11, SG11, and SR11. The amino acids are represented by three-letter codes, with the motif sequence highlighted in red and shown with its respective chemical structure. The 3D peptide structures were generated using the I-TASSER website (https://zhanggroup.org/I-TASSER/, accessed on 5 June 2024). Created with BioRender.com.

The peptides were designed using the cell-penetrating motif (LFVCR), identified in a previous study via MD simulations (GROMACS version 2019.3 software). This motif was originally derived from a non-reported peptide RD10 (RTLFVCRVGD) discovered through deep learning techniques (Ruiz Puentes et al., 2022). To assess the impact of motif positioning on cell penetration efficiency, three peptides were designed with the motif located at the N-terminal, middle, and C-terminal positions within the final sequences. Graphical representations of these designed peptides and the comparative peptides (RD10, MS12, and BUFII) are provided in Figure 1 and Supplementary Figure S1, respectively.

The physicochemical parameters of the designed peptides were evaluated using different tools. Molecular weight (MW), theoretical isoelectric point (pI), and GRAVY were estimated using ProtParam, available on the bioinformatics resource portal ExPASy of the Swiss Institute of Bioinformatics website (http://web.expasy.org/protparam, accessed on 5 June 2024). Net charge at pH 7.0 was evaluated using PepCalc (https://pepcalc.com/, accessed on 5 June 2024). The Boman index was determined using APD3 (https://aps.unmc.edu/prediction, accessed on 5 June 2024). Hydrophobic moment (μH), and hydrophobicity (H) were estimated using Heliquest (https://heliquest.ipmc.cnrs.fr/cgi-bin/ComputParams.py, accessed on 5 June 2024).

### Synthesis and functionalization of magnetite nanoparticles

Magnetite nanoparticles were synthesized using the chemical co-precipitation method (Cifuentes et al., 2023). Initially, 0.01 mol of FeCl_2_ (Iron (II) chloride tetrahydrate) and 0.02 mol of FeCl_3_ (Iron (III) chloride hexahydrate) were dissolved in 100 mL of Type I water. This solution of iron chlorides was homogenized and then cooled to 2°C. Concurrently, 0.08 mol of NaOH was dissolved in 100 mL of Type I water and cooled to 2°C.The iron chlorides solution was placed in a sealed round-bottom flask, magnetically stirred at 300 rpm, and degassed by bubbling nitrogen to remove oxygen and prevent oxidation. After 10 min, the NaOH solution was added dropwise at a rate of 5 mL/min, with constant stirring and a continuous nitrogen flow. The formation of MNPs was indicated by the appearance of a black precipitate. Following the addition of NaOH, the magnetite solution was stirred at 300 rpm for an additional hour under continuous nitrogen flow. The resultant MNPs were washed three times with a 1.5% (w/v) NaCl solution and twice with Type I water. A neodymium magnet was used to facilitate nanoparticle precipitation between washes, and sonication was applied at a frequency of 40 kHz and an amplitude of 38%.

Post-synthesis, the yield of MNPs was quantified. A 100 mg aliquot of MNPs was resuspended in 40 mL of Type I water and sonicated for 10 min at a frequency of 40 kHz and an amplitude of 38%. Subsequently, 250 µL of TMAH were added, and the solution was sonicated for 1 min under the same parameters and then magnetically stirred for 3 min. This was followed by the addition of 50 µL of glacial acetic acid, with another round of 1-min sonication and 3-min stirring. Finally, 1 mL of APTES (20% v/v) was slowly added to the MNPs solution and allowed to react at 60°C with constant stirring at 200 rpm for 1 h. The silanized nanoparticles were then washed as described previously and stored at 4°C until further use.

### Synthesis of nanobioconjugates

An aliquot of 100 mg of silanized MNPs (MNPs-Si) was dispersed in 40 mL of Type I water and sonicated for 10 min at a frequency of 40 kHz with an amplitude of 38%. Following this, 2 mL of 2% (v/v) glutaraldehyde was added to the MNPs-Si suspension, which was then stirred at 250 rpm for 1 h at 24°C. Subsequently, 100 μL of NH_2_-PEG_12_-NH_2_ (2 mg/mL) was added dropwise and allowed to react under mechanical agitation at 250 rpm for 24 h. The resulting PEGylated MNPs (MNPs-Si-PEG) were washed and stored as described previously.

**FIGURE 2:**
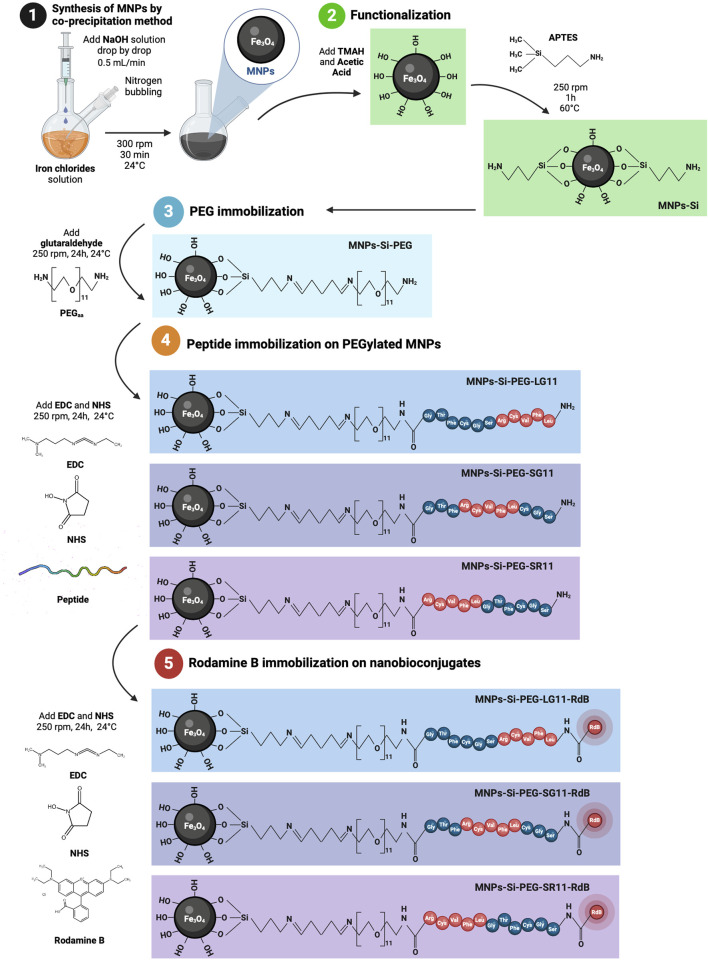
Synthesis of the nanobioconjugates. The process involves: MNP synthesis via the co-precipitation method, APTES functionalization, PEGylation of the functionalized MNPs, immobilization of the designed peptides onto the PEGylated MNPs, and Rhodamine B labeling of the peptide-based nanobioconjugates. Illustrations created with BioRender.com.

The immobilization process aimed to conjugate the carboxyl end of the peptide to the free amine of the PEG, forming an amide bond and allowing to evaluate the motif position. Initially, an aliquot of the peptide was prepared by centrifuging the lyophilized peptide vial for 1 min at 16,000 × *g*, followed by reconstitution in 1 mL of Type I water. Concurrently, 12.3 mg of EDC and 7.4 mg of NHS were dissolved in 10 mL of Type I water. The peptide solution was then added to the EDC/NHS solution and stirred at 250 rpm for 15 min. Subsequently, the EDC/NHS-activated peptide solution was added into the PEGylated MNPs solution and allowed to react for 24 h under mechanical agitation at 250 rpm. The resulting nanobioconjugates were washed and stored as described previously. The synthesis process of the nanobioconjugates is depicted in Figure 2.

### Physicochemical characterizations

The morphology of bare MNPs was examined using transmission electron microscopy (TEM) with a Tecnai F30 instrument (FEI Company, Fremont, CA, United States). The hydrodynamic diameter of the nanoparticles was determined via dynamic light scattering (DLS) analysis using a Zeta-Sizer Nano-ZS (Malvern Panalytical, Malvern, United Kingdom). To prepare for DLS, nanoparticles were diluted in Type I water to a 1% (w/v) concentration, following manufacturer guidelines, and subjected to sonication in an ultrasonic bath to prevent aggregation. Simultaneously, the zeta potential of the nanobioconjugates was measured using the same instrument at room temperature to assess surface charge. DLS and zeta potential measurements were conducted in Type I water at pH 7.4, PBS 1X, DMEM and DMEM supplemented with 10% (v/v) FBS to study aggregation in different solutions. Confirmation of stepwise immobilization on the MNPs was achieved through Fourier transform infrared spectroscopy (FTIR) using a Bruker Alpha II FTIR Eco-ATR (Bruker, Billerica, MA, United States). The absorbance spectra were collected from 4,000 cm⁻^1^–600 cm⁻^1^ with a spectral resolution of 2 cm⁻^1^, providing detailed information on chemical bonds and functional groups. The second derivative of free peptides and nanobioconjugates was calculated using OPUS spectroscopy software to assess changes in the secondary structure of the peptides following immobilization. Thermal stability and conjugation efficiency of the nanoparticles were assessed using thermogravimetric analysis (TGA) performed on a simultaneous TGA/DSC instrument (TA Instruments, New Castle, DE, United States). Each nanobioconjugate sample (approximately 2–8 mg of freeze-dried material) underwent a linear temperature ramp from 25°C to 800°C at a rate of 10°C/min under a nitrogen atmosphere flowing at 100 mL/min. The peptide immobilization efficiency was calculated using Equation 1.

$$Immobilization\;efficiency\;\left(\%\right)=\left[\frac{Peptide\;content}{Total\;amount\;of\;peptide}\right]*100\%$$ (1)

The peptide content was determined based on TGA results (Fadnavis et al., 2003), calculated as the difference in third-step weight loss between the nanobioconjugates and the PEGylated-MNPs. The total amount of peptide refers to the amount initially added during the immobilization process.

### Biocompatibility studies

#### Hemolysis

Hemocompatibility was assessed following the ISO 10993–4:2018 (International Organization for Standardization, 2018). Blood samples were freshly collected from a healthy human donor into a vacutainer tube containing EDTA, with permission granted by the ethics committee at Universidad de Los Andes (minute number 928–2018). Erythrocytes were isolated by centrifugation at 1800 rpm for 5 min, followed by removal of the plasma phase. Erythrocytes were washed with PBS 1X three times. To form a stock solution, 2 mL of the washed erythrocytes (4.5 × 10^6^ erythrocytes/μL) were suspended in 18 mL of PBS 1X and carefully homogenized. The nanobioconjugates were tested at serial dilutions ranging from 100 µg/mL to 6.25 µg/mL in PBS 1X, with Triton X-100 (10% v/v) and PBS 1X serving as positive and negative controls, respectively. For hemolytic activity evaluation, 100 µL of each treatment was mixed with 100 µL of the erythrocyte stock solution in a 96-well microplate. After incubation at 37°C with 5% CO_2_ for 1 h, the plate was centrifuged at 1800 rpm for 5 min. Subsequently, 100 µL of the supernatant was transferred to another 96-well microplate, and the absorbance was measured at 450 nm using a microplate reader. Hemolysis percentage was calculated according to Equation 2.

$$Hemolysis\;\left(\%\right)=\left[\frac{Abs\left(s\right)-Abs\left(-\right)}{Abs\left(+\right)-Abs\left(-\right)}\right]*100\%$$ (2)

Here, $Abs\left(s\right)$ represents the absorbance of the sample, $Abs\left(-\right)$ represents the absorbance of the negative control, and $Abs\left(+\right)$ represents the absorbance of the positive control.

#### Platelet aggregation assay

The platelet aggregation assay was conducted in accordance with the ISO 10993–4:2018 standard (International Organization for Standardization, 2018). Blood samples were freshly collected from a healthy human donor into a vacutainer tube containing sodium citrate, with approval from the ethics committee at Universidad de Los Andes (minute number 928–2018). The blood was centrifuged at 1,000 rpm for 20 min at room temperature to obtain Platelet-rich plasma (PRP). The supernatant containing PRP was collected for use in the assay. Nanobioconjugates were tested at serial dilutions ranging from 100 µg/mL to 6.25 µg/mL. Thrombin (9U) served as the positive control, while PBS 1X was used as the negative control, to compare the aggregation of the platelets. Platelet aggregation potential was assessed by mixing 50 µL of PRP with 50 µL of the respective treatment dilutions in a 96-well microplate. After incubating at 37°C for 5 min, absorbance was measured at 620 nm using a microplate reader. The percentage of platelet aggregation was calculated according to Equation 3.

$$Platelet\;aggregation\;\left(\%\right)=\left[\frac{Abs\left(s\right)-Abs\left(b\right)}{Abs\left(+\right)}\right]*100\%$$ (3)

A standard curve was assessed, and the absorbance of the sample was normalized relative to the absorbance of the nanobioconjugates at 620 nm. Here, $Abs\left(s\right)$ represents the absorbance of the sample, $Abs\left(b\right)$ represents the absorbance of the nanobioconjugates, and $Abs\left(+\right)$ represents the absorbance of the positive control.

#### Cytotoxicity

The cytotoxicity of the nanobioconjugates was evaluated on Vero cells (ATCC^®^ CCL-81) by measuring the metabolic activity associated with the conversion of MTT to formazan. The ISO 10993–5:2009 standard was followed (ISO, 2009). The nanobioconjugates were tested across serial dilutions ranging from 100 µg/mL to 6.25 µg/mL. For the assay, 100 µL of a cell stock solution (1 × 10⁴ cells/well) in DMEM medium supplemented with 5% (v/v) FBS was added to a 96-well microplate and incubated at 37°C with 5% CO_2_ for 24 h. After the initial 24-h incubation, the supplemented DMEM medium was replaced with non-supplemented DMEM medium containing the different concentrations of nanobioconjugates. Cell viability was assessed at 24 and 48-h intervals post-incubation at 37°C, 5% CO_2_. Following the same methodology described above, the cytotoxic effects of labeled and unlabeled nanobioconjugates were compared by evaluating both at a concentration of 25 µg/mL after 0.5 and 4 h of exposure. This assessment was conducted to determine any impact of the labeled nanobioconjugates on cell viability during cellular internalization and endosomal escape assays. For the MTT assay, 10 µL of MTT (5 mg/mL) was added to each well and left to react for 2 h. Then, the culture media were replaced with 100 µL of DMSO to dissolve the formazan crystals formed. Absorbance was measured at 595 nm using a microplate reader. Cell viability was calculated following Equation 4.

$$Cell\;viability\;\left(\%\right)=\left[\frac{Abs\left(s\right)-Abs\left(t\right)}{Abs\left(t\right)-Abs\left(c\right)}\right]*100\%$$ (4)

Here, $Abs\left(s\right)$ represents the absorbance of the sample, $Abs\left(t\right)$ represents the absorbance of the cells exposed to 1% (v/v)-Triton X-100, and $Abs\left(c\right)$ represents the absorbance of the cells that were not exposed to any treatment.

#### Procoagulant activity

The procoagulant activity of the nanoparticles was assessed following protocols from previous studies (Avsievich et al., 2019; Bian et al., 2019). Fresh blood samples were collected from a healthy human donor into EDTA-containing vacutainer tubes, with ethical approval from the Universidad de Los Andes (approval number 928–2018). After centrifugation at 2,500 rpm for 10 min, platelet-rich plasma and buffy coat were removed by aspiration. The erythrocytes were washed three times with PBS 1X, and 8 µL of the washed erythrocytes were added to 1 mL of nanoparticle solution (100 µg/mL) prepared in PBS 1X. Samples were incubated for 2 h at 25°C and centrifuged again under the same conditions. To prevent erythrocyte aggregation and facilitate single-cell measurements, the erythrocytes with nanobioconjugates were collected from the bottom of the tube and diluted in platelet-poor plasma (PPP) at a ratio of 1:10 µL (RBCs: PPP). Erythrocyte aggregation was analyzed at a multicellular level by capturing light microscopy images using a Zeiss™ Primo Star Microscope (Zeiss, Germany) with a 40X/0.65 objective. Single cells and aggregates were quantified using Fiji-ImageJ^®^ software.

To obtain PPP (Avsievich et al., 2019), fresh blood samples were collected as described above and centrifuged at 3,000 × *g* for 10 min. The supernatant was collected and centrifuged again under the same conditions, with the resulting supernatant serving as the PPP.

### Labeling of nanobioconjugates with Rhodamine B

To facilitate the tracking of nanobioconjugates during confocal microscopy analyses, they were labeled with Rhodamine B. Initially, 12.3 mg of EDC and 7.4 mg of NHS were dissolved in 5 mL of Type I water. Subsequently, 5 mg of Rhodamine B was added to the solution and allowed to react under constant stirring (250 rpm) in a dark room for 15 min at 40°C to activate the carboxyl groups. This Rhodamine B solution was then added to 100 mg of each nanobioconjugate (previously resuspended in 40 mL of Type I water) and left to react for 24 h under mechanical agitation (250 rpm), covered in aluminum foil to protect from light. After labeling, the nanoparticles were washed with a 1.5% (w/v) NaCl solution until no Rhodamine B remained in the supernatant, followed by two washes with Type I water to remove excess salts, aided by a neodymium magnet. The labeled nanoparticles were stored at 4°C in complete darkness until further use.

### Cellular internalization pathways and endosomal escape analysis

The cellular uptake and endosomal escape of the nanobioconjugates were investigated using confocal microscopy in Vero cells. Cells were resuspended in DMEM medium supplemented with 5% (v/v) FBS. A stock solution with a density of 150,000 cells/mL was prepared, and 100 µL was added to glass slides pre-treated with poly-D lysine. After a 24-h incubation period to allow for cell attachment, the medium was replaced with the route inhibitor. Dynasore hydrate was used to inhibit clathrin-mediated endocytosis (McCluskey et al., 2013; Preta et al., 2015). Specifically, Dynasore inhibits the GTPase activity of dynamin1, dynamin2, and Drp1, the mitochondrial dynamin. Additionally, it may interfere with cholesterol homeostasis and actin dynamics (Macia et al., 2006; Preta et al., 2015). This inhibitor was prepared at a concentration of 150 µM (Piccini et al., 2015) in non-supplemented DMEM medium.

Two sets of experiments were conducted: one with the clathrin pathway inhibitor and the other without inhibitor. For the inhibition experiment, the Dynasore solution was added to the glass slides and incubated under a humidified atmosphere at 37°C with 5% CO_2_ for 30 min. Subsequently, cells were exposed to Rhodamine B-labeled nanobioconjugates in non-supplemented DMEM medium at a concentration of 25 µg/mL for 30 min and 4 h. For the test without the inhibitor, cells were exposed to the nanobioconjugate solution and evaluated under the same conditions. After exposure, cells were washed with PBS 1X and stained with Hoechst 33342 (1:10,000) and Lysotracker Green DND-26 (1:10,000) in non-supplemented medium for 5 min to label nuclei and endosomes, respectively. Following exposure, cells were washed with PBS 1X and then stained with Hoechst 33342 (1:10,000) and Lysotracker Green DND-26 (1:10,000) in non-supplemented medium for 5 min to label the nuclei and endosomes, respectively. Images were captured using an Olympus FV1000 confocal laser scanning microscope (CLSM) (Olympus, Japan) equipped with a 20X/0.75 UPlanSApo and a 40X/0.6 UCPlan FL N objetive. Excitation/emission wavelengths of 358 nm/461 nm, 488 nm/520 nm, and 546 nm/575 nm were used to detect nuclei, endosomes, and Rhodamine B-labeled nanobioconjugates, respectively. The analysis involved capturing 10 images for each treatment (10 cells per image). Image analysis was performed using Fiji-ImageJ^®^ software to calculate colocalization and the percentage of the area covered by the nanobioconjugates.

### Statistical analysis

All data measurements are presented as mean ± standard deviation from experiments conducted in triplicate. Statistical analysis was performed using GraphPad Prism 10.2.1 software (San Diego, CA, United States). Statistical comparisons were conducted using one-way or two-way ANOVA followed by Sidak’s or Tukey’s multiple comparisons test, as appropriate. Results were considered statistically significant at a p-value ≤ 0.05 (*), where * denotes a significant difference with a p-value in the range of 0.01 ≤ p < 0.05, ** for 0.001 ≤ p < 0.01, *** for 0.0001 ≤ p < 0.001, and **** for p < 0.0001.

### Generative AI use

Generative AI technology, specifically OpenAI’s ChatGPT (version GPT-4, model “gpt-4-turbo-16k”), was used to enhance the text by suggesting revisions for clarity and conciseness. The authors reviewed and approved all edits to ensure the content’s accuracy and alignment with the research objectives.

## Results and discussion

### Physicochemical properties of the peptides

The physicochemical properties of the designed peptide sequences are summarized in Table 1. The designed peptides LG11, SG11 and SR11 had a net positive charge at physiological pH. Glycosaminoglycans (GAGs) and proteoglycans are critical molecules that are exposed at the outer surface of the lipid bilayer and important for CPP cell entry. Particularly, heparan sulfate and other sulfated GAGs attract cationic CPPs by their negative charges, thus acting as primary binding site for CPPs and subsequent internalization of the peptide (Neundorf, 2019). Hydrophilic peptides typically have a low or negative GRAVY score, while hydrophobic peptides have a high positive GRAVY score (Kyte and Doolittle, 1982). The positive GRAVY scores for LG11, SG11, and SR11 indicate that these peptides are hydrophobic. Hydrophobic peptides are more likely to interact with the lipid bilayer of cell membranes, which can facilitate cell penetration through direct translocation mechanisms rather than energy-dependent pathways (Gomez et al., 2010). The peptides also exhibit high hydrophobicity and a high isoelectric point (pI). An *in silico* study showed that short peptides with low hydrophobicity and low pI are tended to have the lowest uptake (Matsumoto et al., 2015). Therefore, the high hydrophobicity and high pI values of these peptides suggest effective cell uptake, as hydrophobic interactions play a significant role in crossing cell membranes.

**TABLE 1:** Designed peptides physicochemical properties.

Residues	MW (g/mol)	Net charge at pH 7	GRAVY	Theoretical pI	Hydrophobicity (H)	Hydrophobic moment (μH)	Boman Index
11	1,189.42	+0.91	1.073	8.07	0.799	0.182	0.13

The hydrophobic moment (μH) of a peptide measures its amphipathicity and is an important factor in peptide-mediated membrane disturbance. Peptide-induced membrane leakage tends to be proportional to μH. Therefore, minimizing μH is desirable to reduce CPP-induced plasma membrane damage (Hällbrink et al., 2001). Peptides with high μH (∼0.391 and above) show high lytic activity, while those with a low μH (∼0.272 and below) exhibit minimal cytotoxicity (Chen et al., 2017). The low μH values for LG11, SG11, and SR11 indicate that these peptides are less likely to cause membrane disturbance or cytotoxicity, which is beneficial for maintaining cell viability while ensuring efficient internalization. The Boman index represents the potential of a peptide to bind to membranes or other proteins. A high Boman index (>2.48) indicates high binding potential, while a low Boman index (≤1) suggests fewer side effects and lower toxicity to mammalian cells (Boman, 2003). The low Boman index values for these peptides suggest that they are less likely to bind nonspecifically to proteins, reducing the risk of off-target effects. These properties enhance the safety and specificity of the peptides for biomedical applications.

### Physicochemical characterization of the nanobioconjugates

**FIGURE 3:**
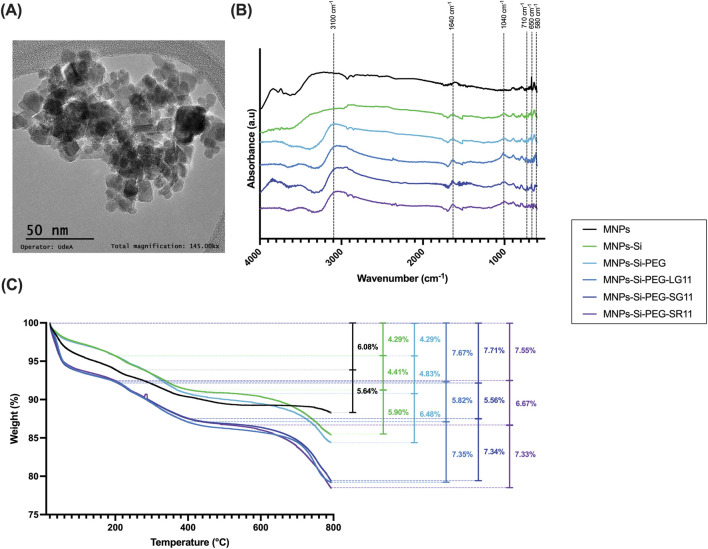
Physicochemical characterization of the nanobioconjugates. **(A)** Microscopic characterization of bare MNPs analyzed through TEM at 145 kX, **(B)** FTIR spectra, and **(C)** TGA analysis.

Physicochemical characterizations of the evaluated nanobioconjugates are presented in Figure 3, while results for the comparative nanobioconjugates with peptides RD10, MS12, and BUFII can be found in the Supplementary Figure S2. Among the parameters influencing the efficacy of nanoparticle-based bioactive molecule delivery systems, nanoparticle size plays a crucial role in determining their performance *in vitro* and *in vivo* for biomedical applications. Properties affected by nanoparticle size include superparamagnetism, toxicity, protein adsorption on the surface, cellular internalization processes, targeted drug delivery, biodistribution, surface reactivity, and tissue marking (Ortega and Reguera, 2019; Montiel Schneider et al., 2022; Petrov and Chubarov, 2022; Nica et al., 2023). TEM was employed to study the morphology and crystalline structure of the bare nanoparticles (Figure 3A). The image illustrates monocrystalline MNPs with uniform sizes and shapes, consistent with previous studies on magnetic nanoparticles (Deng et al., 2005; Krispin et al., 2012; Nguyen et al., 2021).

Surface modifications of magnetite nanoparticles were confirmed using Fourier transform infrared (FTIR) spectroscopy. Figure 3B and Supplementary Figure S2A show the FTIR spectra for the bare MNPs and all the nanobioconjugates. Common peaks at 580 cm⁻^1^ and 650 cm⁻^1^ across all spectra can be attributed to the vibration of the Fe-O bond in iron oxide (Cuellar et al., 2018; Perez et al., 2019; Lopez-Barbosa et al., 2020; Rangel-Muñoz et al., 2020). Silanization was confirmed by the presence of the Si-O stretching vibration at 1,040 cm⁻^1^ and the C-H bending peak at 710 cm⁻^1^, indicating successful functionalization with APTES (Cuellar et al., 2018; Perez et al., 2019; Lopez-Barbosa et al., 2020; Rangel-Muñoz et al., 2020). During the PEGylation process, glutaraldehyde was used as a crosslinker, forming imide bonds with the silanized nanoparticles and the PEG molecule (see step 3 of Figure 2). The strong absorption band at 3,100 cm⁻^1^ corresponds to N–H stretching vibrations associated with amines (Eckel et al., 2001; Ferreira et al., 2022). For peptide immobilization, the zero-length crosslinkers EDC and NHS were employed to form an amide bond between the amino end of the PEGylated nanoparticles and the C-terminal of the peptide (see step 4 of Figure 2). The peak observed at 1,640 cm⁻^1^ corresponds to amide I, confirming the successful immobilization of the peptides on the nanoparticle surface (Cuellar et al., 2018; Perez et al., 2019).

The secondary structure of the peptides with the motif and the comparative peptides was predicted using PEP-FOLD3 (Thevenet et al., 2012; Shen et al., 2014; Lamiable et al., 2016) (Supplementary Figures S3A, C, E, G, I, K). To ensure that immobilization on nanoparticles did not induce significant structural changes in the peptide, the second derivative of FTIR was analyzed between the wavelengths 1,550 cm⁻^1^ and 1750 cm⁻^1^ (blue box) (Supplementary Figures S3B, D, F, H, J, L). These peptide predictions guided the analysis of the second derivative and the determination of the secondary structures of both the free peptides and the peptides after immobilization. LG11 exhibited random coil structures in the center (C4 to G7) observed at 1,651 cm⁻^1^ (Barth, 2007; Kong and Yu, 2007; Lewis et al., 2013) and beta-sheet structures at the ends, observed at 1,696 cm⁻^1^ (Kumosinski and Farrell, 1993; Jackson and Mantsch, 1995; Kong and Yu, 2007; Lewis et al., 2013; De Meutter and Goormaghtigh, 2021). After immobilization, the peak for the random coils shifted to 1,655 cm⁻^1^, while the beta-sheet peak remained unchanged. SG11 displayed random coil structures throughout its sequence, observed at 1,650 cm⁻^1^ (Barth, 2007; Kong and Yu, 2007; Lewis et al., 2013). After immobilization, this peak shifted to 1,656 cm⁻^1^. SR11 exhibited random coil structures at the ends (S1 to G2 and L7 to C10), observed at 1,676 cm⁻^1^ (Barth, 2007; Kong and Yu, 2007; Lewis et al., 2013), and an alpha-helix structure in the center (C3 to G6), observed at 1,658 cm⁻^1^ (Jackson and Mantsch, 1995; Barth, 2007; Kong and Yu, 2007). After immobilization, the random coil peak shifted to 1700 cm⁻^1^, while the alpha-helix peak shifted to 1,655 cm⁻^1^.

For the comparative peptides, RD10 showed random coil structures throughout its entire sequence, observed at 1,657 cm⁻^1^ (Barth, 2007; Kong and Yu, 2007; Lewis et al., 2013). After immobilization, this peak remained unchanged. MS12 exhibited alpha-helix structures from F2 to L10, observed at 1,650 cm⁻^1^ (Jackson and Mantsch, 1995; Barth, 2007; Kong and Yu, 2007). After immobilization, the peak shifted to 1,655 cm⁻^1^. Finally, BUFII displayed alpha-helix structures at the ends (R2 to R5 and V12 to L19), observed at 1,650 cm⁻^1^ (Jackson and Mantsch, 1995; Barth, 2007; Kong and Yu, 2007), and random coil structures in the center (A6 to P11), observed at 1,673 cm⁻^1^ and 1,680 cm⁻^1^ (Barth, 2007; Kong and Yu, 2007; Lewis et al., 2013). After immobilization, the alpha-helix peak shifted to 1,658 cm⁻^1^, while the random coil peak shifted to 1,685 cm⁻^1^. Overall, no significant changes were observed in the secondary structures of the peptides after immobilization.

The surface modifications of the MNPs were also assessed via TGA (Figure 3C; Supplementary Figure S2B) to verify the successful immobilization, the immobilization efficiencies of the attached molecules, and the thermal stability of the nanobioconjugates. TGA is widely used in the characterization of biomaterials (Loganathan et al., 2017; Seifi et al., 2020) that can be employed to verify the successful immobilization and efficiency of molecule attachment on the nanoparticle surface (Almaghrabi et al., 2023). For the bare nanoparticles, two weight losses were observed, whereas three weight losses were noted for the nanobioconjugates due to surface functionalization, consistent with other studies (Cuellar et al., 2018; Perez et al., 2019; Lopez-Barbosa et al., 2020; Rangel-Muñoz et al., 2020). The first weight loss, occurring between 24°C and 200°C, ranged from 4.29% to 7.71% and is associated with the dehydration of the sample (loss of physically adsorbed water on the nanobioconjugate surface). The second weight loss, from 200°C to 400°C, ranged from 4.41% to 10.4% and can be attributed to the desorption of physically adsorbed organic and inorganic compounds that remain from the synthesis and immobilization processes. The third weight loss, occurring from 400°C to 800°C, ranged from 5.90% to 7.35% and corresponds to the detachment of the APTES, PEG, and peptide molecules from the nanoparticle. Supplementary Table S1 summarizes the peptide immobilization efficiencies for each nanobioconjugate, with all efficiencies exceeding 50%. Overall, the synthesized nanobioconjugates demonstrate substantial thermal stability below 400°C, indicating their safety at physiological temperatures.

**FIGURE 4:**
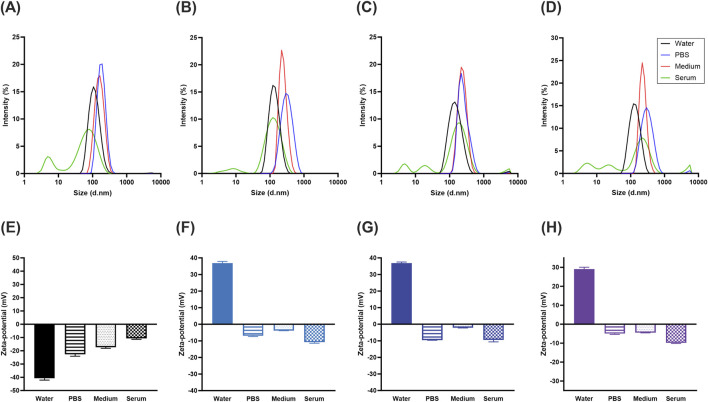
DLS intensity plots of comparative nanobioconjugates dispersed in water at pH 7.4 (water), PBS 1X (PBS), DMEM (medium), and DMEM + 10% (v/v) FBS (serum) for the **(A)** bare MNPs, **(B)** LG11, **(C)** SG11, and **(D)** SR11 nanobioconjugates. Zeta potential measurements of the nanobioconjugates under the same conditions for the **(E)** bare MNPs, **(F)** LG11, **(G)** SG11, and **(H)** SR11 nanobioconjugates.

Figures 4A–D and Table 2 show the average hydrodynamic diameters of each nanobioconjugate in various media, while Supplementary Figures S4A–C and Supplementary Table S2 present the data for the comparative nanobioconjugates. In water, the bare MNPs exhibited an average hydrodynamic diameter of 109.9 ± 1.106 nm, while both the designed and comparative nanobioconjugates had average diameters below 160 nm. The results demonstrate a progressive increase in hydrodynamic diameter with each functionalization step, including silanization, PEGylation, and peptide immobilization.

**TABLE 2:** DLS and zeta potential test results for the nanobioconjugates, showing nanoparticle size, polydispersity index, and surface charge in water at pH 7.4 (water), PBS 1X (PBS), DMEM (medium), and DMEM + 10% (v/v) FBS (serum).

Solution	Nanobioconjugate	Average diameter (nm)	Polydispersity index	Zeta-potential (mV)
Water	MNPs	109.9 ± 1.106	0.168 ± 0.017	−40.8 ± 1.350
	MNPs-Si-PEG	120.8 ± 1.464	0.149 ± 0.001	29.70 ± 0.777
	MNPs-Si-PEG-LG11	128.2 ± 0.656	0.141 ± 0.029	36.80 ± 1.10
	MNPs-Si-PEG-SG11	137.5 ± 0.322	0.166 ± 0.019	36.93 ± 0.643
	MNPs-Si-PEG-SR11	126.0 ± 1.550	0.133 ± 0.003	29.08 ± 0.977
PBS	MNPs	198.0 ± 17.76	0.235 ± 0.026	−22.7 ± 1.44
	MNPs-Si-PEG-LG11	298.6 ± 28.53	0.149 ± 0.032	−6.95 ± 0.437
	MNPs-Si-PEG-SG11	276.4 ± 6.766	0.235 ± 0.085	−9.62 ± 0.0666
	MNPs-Si-PEG-SR11	294.8 ± 22.79	0.160 ± 0.037	−4.96 ± 0.405
Medium	MNPs	160.7 ± 20.02	0.205 ± 0.014	−17.3 ± 0.839
	MNPs-Si-PEG-LG11	272.0 ± 12.16	0.236 ± 0 .038	−3.78 ± 0.111
	MNPs-Si-PEG-SG11	244.3 ± 18.25	0.194 ± 0.025	−2.135 0.134
	MNPs-Si-PEG-SR11	300.5 ± 3.180	0.319 ± 0.035	−4.50 ± 0.0608
Serum	MNPs	83.53 ± 1.328^a^	0.653 ± 0.055	−10.7 ± 0.681
		6.953 ± 2.795^b^		
	MNPs-Si-PEG-LG11	141.4 ± 5.380^a^	0.534 ± 0.051	−10.8 ± 0.600
		9.642 ± 3.125^b^		
	MNPs-Si-PEG-SG11	211.1 ± 9.292^a^	0.724 ± 0.005	−9.52 1.11
		8.939 ± 6.453^b^		
	MNPs-Si-PEG-SR11	233.4 ± 8.584^a^	0.930 ± 0.122	−9.87 ± 0.302
		7.0245 ± 2.776^b^		

^a^ Refers to the first (maximum) peak.

^b^ Refers to the second peak.

In water, all nanobioconjugates exhibited smaller diameters compared to those in PBS and medium, likely due to water providing a more stable environment that enhances the nanoparticle surface charge (Wiogo et al., 2011). Conversely, the high ionic strength of PBS and medium suppresses the electric double layer and reduces electrostatic repulsion, promoting nanoparticle agglomeration (Wiogo et al., 2011; Mbeh et al., 2015). Interestingly, although medium has a higher salt content than PBS, which would typically result in greater agglomeration, most nanobioconjugates exhibited a size reduction when transitioning from PBS to medium. In serum, multiple peaks were observed, likely representing agglomerates of various sizes and the coexistence of protein monomers and aggregates. The presence of smaller nanoparticle agglomerates may be attributed to structural interactions between the immobilized peptides and serum proteins, which bind to the peptide surfaces and stabilize the nanoparticles—a phenomenon known as the protein corona effect (Mbeh et al., 2015). Serum components such as globulin and transferrin are known to interact with nanoparticles, helping prevent further agglomeration (Ji et al., 2010; Yu et al., 2014).

The polydispersity index (PDI) is a dimensionless number used to quantify the uniformity of size distribution among molecules or particles in a sample. PDI values above 0.7 typically indicate high polydispersity, making the sample unsuitable for analysis by DLS (Danaei et al., 2018). In contrast, PDI values below 0.2 are considered optimal for polymer-based nanomaterials (Lahiri et al., 2017; Danaei et al., 2018). In this study, higher PDI values (above 0.2) were primarily observed in nanobioconjugates dispersed in medium and serum. The elevated PDI in serum, along with a broad particle size distribution, can be attributed to the coexistence of Bovine Serum Albumin (BSA) monomers and agglomerates (Orts-Gil et al., 2011; Hondow et al., 2012). All nanobioconjugates evaluated in water exhibited PDI values below 0.185, while most nanobioconjugates in PBS and medium had PDI values under 0.2, indicating a narrow size distribution and good nanoparticle homogeneity. Interestingly, it was observed that when the surface charge approached neutrality, the hydrodynamic size increased due to agglomeration (Mbeh et al., 2015). Maintaining low PDI values is crucial for ensuring consistent physical and chemical properties, reproducibility, stability, and efficacy of nanocarriers. These characteristics suggest a wide range of potential applications in the biomedical field, including drug delivery (Anik et al., 2021), cellular internalization processes (Zhao et al., 2011), intravenous injection (Kudr et al., 2017), targeted therapy (Fatima et al., 2021), biosensors (Rocha-Santos, 2014), and others.

The zeta potential is a physical property that allows for the determination of the surface charge of particles and quantify the magnitude of the electrostatic repulsion or attraction between molecules in a suspension. It provides valuable insights into the long-term stability of colloidal systems by indicating whether molecules are likely to disperse, aggregate, or flocculate (Zavisova et al., 2019). Large zeta potentials are generally defined as values greater than +25 mV or less than −25 mV, indicating that the molecules tend to repel each other and avoid agglomeration, coagulation or flocculation (Bhattacharjee, 2016; Zavisova et al., 2019). Figures 4E–H and Table 2 show the zeta potential measurements obtained for nanobioconjugates dispersed in different media. For the MNPs in water, a negative surface charge of −40.8 ± 1.350 mV was observed, consistent with values reported (Li et al., 2022). After the PEGylation of the nanoparticles, the surface charge shifted to positive, with a value of +29.70 ± 0.777 mV. This change in surface charge is consistent with previous studies confirming that PEG is a polycationic polymer (Chen et al., 2013). After peptide immobilization, the zeta potentials for the LG11-, SG11-, and SR11-nanobioconjugates were +36.80 ± 1.10 mV, +36.93 ± 0.643 mV, and +29.08 ± 0.977 mV, respectively. These results indicate that the nanobioconjugates in water achieved optimal zeta potential values, promoting molecular repulsion and system stability.

In contrast, in solutions such as PBS, medium, and serum, the zeta potentials of all nanobioconjugates and bare MNPs were negative, consistent with previous studies (Chircov et al., 2023; Portilla et al., 2023). The negative charge of the nanobioconjugates and bare MNPs in PBS can be attributed to the adsorption of phosphate groups (HPO₄^2^⁻/H₂PO₄⁻) and bicarbonate (HCO₃⁻) onto the nanoparticle surfaces (Kaewsichan et al., 2011; Zhu et al., 2020). Similarly, in medium, anions such as sulfates (SO₄^2^⁻), bicarbonate, and phosphate groups adsorb onto the particles, imparting a negative charge (Harjo et al., 2019; Bița et al., 2022). In serum, the same anions are present, along with BSA, which is negatively charged at physiological pH. The interaction and adsorption of BSA onto the nanoparticle surfaces further explain the negative charge of the nanobioconjugates in serum (Fologea et al., 2007). Compared to water, the zeta potentials in PBS, medium, and serum were generally lower due to the higher ionic strength of these solutions, which reduces the thickness of the electric double layer, decreases electrostatic repulsion, and facilitates particle interactions (Choi et al., 2004). The differences in zeta potential between medium and serum likely arise from the varying protein content in these solutions, with BSA adsorption altering both the surface charge and zeta potential of the nanoparticles. A similar trend was observed for the comparative nanobioconjugates of peptides RD10, MS12, and BUFII, with positive zeta potentials in water and negative zeta potentials in PBS, medium, and serum. These results are presented in Supplementary Figures S4D–F and Supplementary Table S2.

### Biocompatibility studies

**FIGURE 5:**
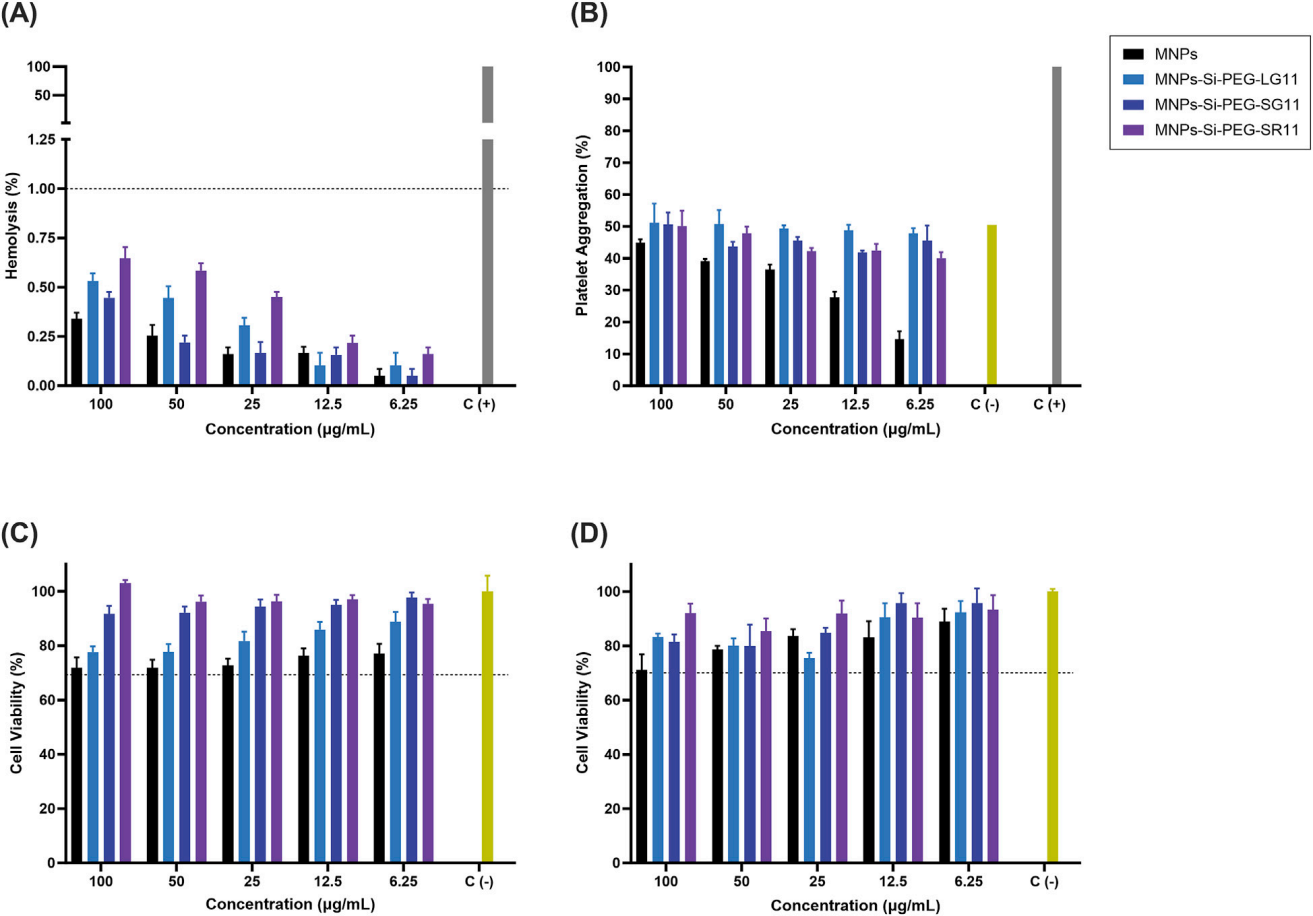
Biocompatibility and cell viability of nanobioconjugates. **(A)** Hemolytic effect assessment, using PBS 1X as the negative control and Triton X-100 (10% v/v) as the positive control. **(B)** Platelet aggregation analysis, with PBS 1X as the negative control and Thrombin (9U) as the positive control. Cell viability of Vero cells after nanobioconjugate exposure for **(C)** 24 h and **(D)** 48 h. Non-treated cells were employed as positive control.

Figures 5A, B illustrate the hemolytic activity and platelet aggregation effects of the nanobioconjugates, respectively. All treatments exhibited average hemolytic activity below 1%, and no significant platelet aggregation was observed compared to the PBS control. Generally, the nanobioconjugates showed an increase in platelet aggregation compared to the bare MNPs, with the LG11-nanobioconjugate displaying slightly more aggregation among the designed nanobioconjugates. Additionally, a platelet uptake assay was developed to assess whether nanoparticles were internalized by platelets (see Supplementary Figure S5). The results indicated that peptide immobilization slightly enhanced nanoparticle uptake by platelets. MNPs showed an uptake of 6.12%, while the LG11-, SG11-, and SR11-nanobioconjugates exhibited uptakes of 12.76%, 14.27%, and 13.84%, respectively. Although the nanobioconjugates demonstrated increased platelet uptake, these differences were not statistically significant.

Previous studies have shown that CPPs can activate surface receptors on platelet membranes, particularly at higher concentrations where this mechanism is amplified (Ruseska and Zimmer, 2020). This suggests that the LFVCR motif may contain amino acids that influence this process. Arginine, a precursor to nitric oxide (NO), has been reported to play a dual role depending on the local environment, either inhibiting platelet aggregation via NO production or supporting aggregation through other pathways (Gambaryan and Tsikas, 2015; Gawrys et al., 2020). It is hypothesized that in the LG11-nanobioconjugate, arginine might be more exposed and interact more with platelets, leading to the slight increase in platelet aggregation observed. However, the overall influence of the LFVCR motif on platelet aggregation appears to be minimal. Other amino acids in the motif, such as leucine, phenylalanine, and valine, have been studied for their anticoagulant effects (Xu et al., 2020; Grigorieva et al., 2023; Qin et al., 2023). Additionally, cysteine residues might influence antiplatelet adhesion through the HNP-1 pathway, though their exact role under normal physiological conditions remains unclear (McDaniel et al., 2019). Overall, the results indicate that the nanobioconjugates are suitable for intravenous administration with a low risk of causing thrombosis. According to the ISO 10993–4:2018 standard, these findings underscore the high hemocompatibility of the nanobioconjugates (International Organization for Standardization, 2018).

Figures 5C, D show the cytocompatibility results of the nanobioconjugates on Vero cells over 24 and 48 h of exposure, respectively. The results indicate that the nanobioconjugates exhibited significantly higher cell viability compared to the bare MNPs, suggesting that the peptides contribute substantially to the biocompatibility of the MNPs. These findings align with the predictions of the peptides’ physicochemical properties. The low μH values predicted for the designed peptides indicated that these peptides are less likely to cause membrane disturbance or cytotoxicity (Hällbrink et al., 2001), a characteristic confirmed by the cytocompatibility results. For both exposure times, the LG11-nanobioconjugate exhibited the lowest cell viability percentages compared to the other nanobioconjugates. This reduction in cell viability can be attributed to a high uptake of the nanobioconjugate by the cell line (Cuellar et al., 2018; Perez et al., 2019; Cifuentes et al., 2023). This result will be analyzed further in the next section. Overall, all vehicles showed cell viability percentages above 70%, qualifying them as non-cytotoxic according to the ISO 10993–5:2009 standard (ISO, 2009). These findings confirm the excellent biocompatibility of the nanobioconjugates and highlight their potential for safe cellular uptake.

The cytotoxic effects of Rhodamine B-labeled nanobioconjugates were assessed at 0.5 and 4 h, with comparisons to unlabeled nanobioconjugates to evaluate their impact on cell viability in the cellular internalization assays (Supplementary Figure S6). The results showed that labeling with Rhodamine B reduce the viability in Vero cells at both intervals. At the 0.5-h mark, viability decreased in a range of 3.29%–20.43%, and at the 4-h mark, viability decreased in a range of 7.61%–19.05%. The cytotoxic effects observed are consistent with the known properties of this fluorescent dye, which affects various cell lines, including Vero cells (Serbian et al., 2020; Shi et al., 2020). Despite these effects, cell viability of labeled nanobioconjugates remained above 70%.

**FIGURE 6:**
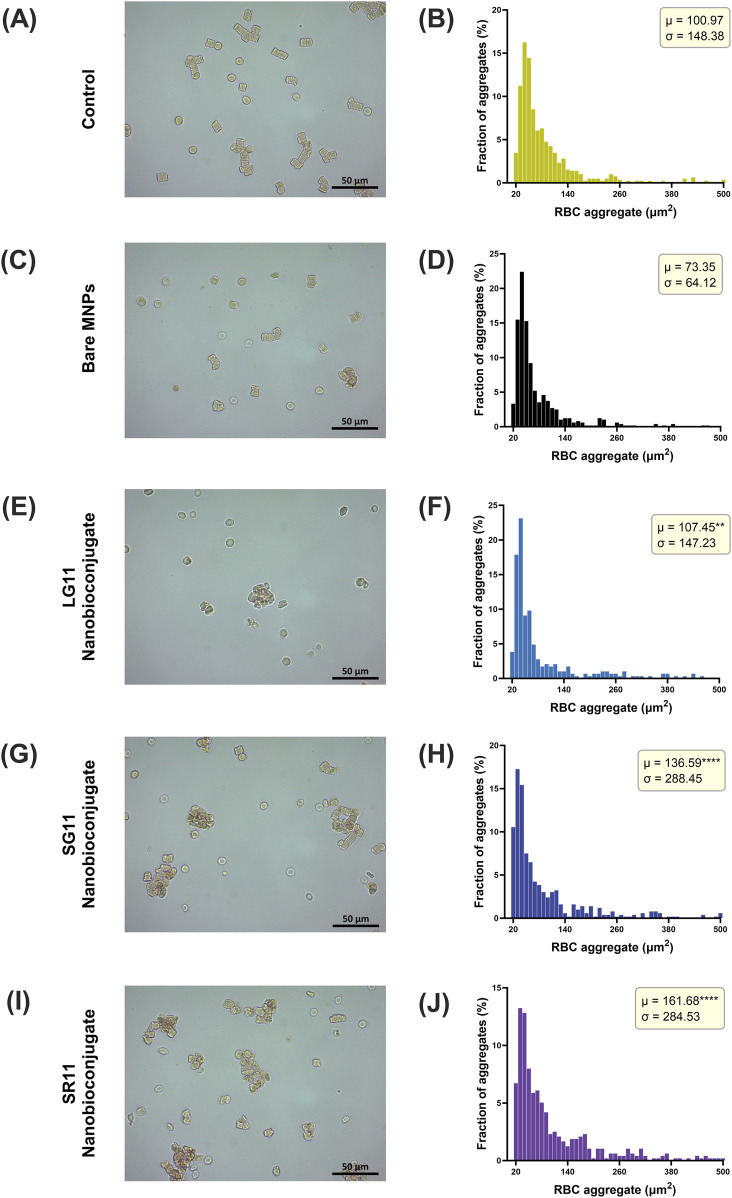
Relative size distribution of RBC aggregates observed at 40× magnification using conventional optical microscopy under **(A)** normal conditions and upon exposure to **(C)** Bare MNPs, **(E)** LG11, **(G)** SG11, and **(I)** SR11 nanobioconjugates. Corresponding size distribution histograms are provided for (B) normal conditions and for exposure to **(D)** Bare MNPs, **(F)** LG11, **(H)** SG11, and **(J)** SR11 nanobioconjugates. The symbol * corresponds to a statistically significant difference with a p-value in the range of 0.01 ≤ p-value ≤ 0.05, ** to a statistically significant difference with a p-value in the range of 0.001 ≤ p-value <0.01, *** to p-value in the range of 0.0001 ≤ p-value ≤ 0.001 and **** to p-value < 0.0001.

Figure 6 illustrates the evaluation of the procoagulant activity of bare MNPs and nanobioconjugates. The general results reveal that erythrocytes (RBCs) incubated with nanobioconjugates tend to form aggregates of irregular size and shape, distinct from those observed in the control and bare MNPs, which exhibited smaller agglomerations. In the control sample and in the presence of bare MNPs, RBCs formed typical rouleaux-shaped aggregates (Figures 6A, C). This phenomenon occurs because RBCs tend to stick together in plasma due to proteins like fibrinogen, which reduce the repulsive forces between cells, allowing RBC stacking (Rampling, 1999; Avsievich et al., 2019). However, these characteristic forms were reduced in RBCs exposed to nanobioconjugates, where larger, irregular agglomerates were observed (Figures 6E, G, I). The size distribution of RBC aggregates is depicted in Figures 6B, D, F, H, J, allowing the determination of mean and standard deviation for aggregate sizes. Notably, the LG11-nanobioconjugate resulted in a mean agglomerate size of 107.45 µm^2^, close to that observed under normal conditions (100 μm^2^). In contrast, the other nanobioconjugates significantly increased the size of RBC aggregates, with the SG11-nanobioconjugate showing an average size of 136.59 µm^2^ and the SR11-nanobioconjugate at 161.68 µm^2^. These findings indicate that the peptide with the exposed motif in the LG11-nanobioconjugate induced the least increase in procoagulant activity among the nanobioconjugates. A previous study has demonstrated that MNPs can exhibit procoagulant activity by aggregating and adhering to RBC membranes (Ran et al., 2015). However, in our study, bare MNPs did not show a significant increase in procoagulant activity. A slight increase was observed for the LG11-nanobioconjugate, while a more pronounced increase was found for the SG11 and SR11 nanobioconjugates.

The biocompatibility results compile evidence supporting the nanobioconjugates’ potential use in numerous applications and therapies. Their low thrombotic, hemolytic, and cytotoxic potential, along with their procoagulant activity, highlights their suitability for therapeutic use via intravenous administration (Shariatinia, 2021). The absence of cellular injury risk makes these nanocarriers suitable candidates for intracellular drug delivery, facilitating anticancer therapies, delivering anti-inflammatory drugs, and aiding in diagnostic applications by transporting markers for medical imaging (Derakhshankhah and Jafari, 2018). Additionally, various studies have proven that CPPs can assist in treating Central Nervous System disorders. The blood-brain barrier (BBB) is known for its low permeability, standing a significant challenge for drug delivery to the brain. However, biocompatible CPP-based nanocarriers have demonstrated successful transportation of therapeutic agents across the BBB, enabling effective treatment of neurological conditions (Hersh et al., 2022). This capability to cross the BBB and deliver drugs directly to the brain underscores the potential of CPPs in addressing diseases such as Alzheimer’s, Parkinson’s, and other neurodegenerative disorders, providing a promising avenue for future therapeutic developments (Sharma et al., 2016; Xie et al., 2020).

### Cellular internalization pathways and endosomal escape analysis

Confocal microscopy was employed to investigate the cellular uptake mechanisms of the designed nanobioconjugates and MNPs on the Vero cell line. Understanding these processes is crucial for biomedical applications, as it elucidates how molecules of interest enter cells. The energy-dependent process of cellular uptake, known as endocytosis, involves the internalization of external molecules into the cell through the formation of invaginations in the cell membrane (Mayor and Pagano, 2007; Doherty and McMahon, 2009). These internal structures are known as vesicles (or endosomes). Clathrin-mediated endocytosis is the most common endocytic mechanism in all cell types and tissues due to its high availability and adaptability towards recognizing numerous agents (Rueda-Gensini et al., 2020) and has been extensively documented in previous studies as a prevalent route for nanobioconjugate cellular uptake, including iron oxide nanoparticles (IONPs) (Lunov et al., 2011; Luther et al., 2013; Chen et al., 2017).

**FIGURE 7:**
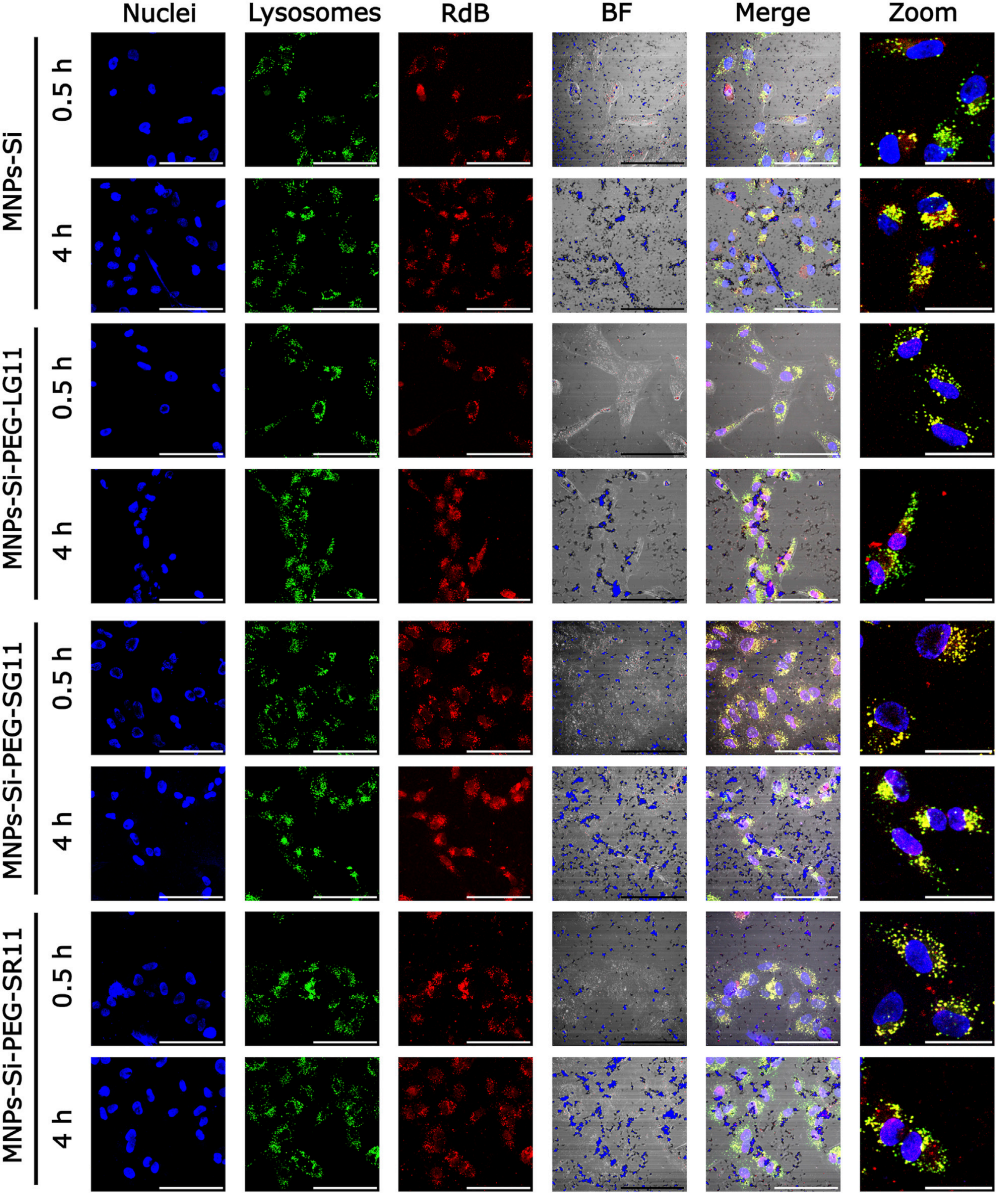
Confocal microscopy images for cell internalization and endosomal escape analysis in Vero cells at 0.5 h and 4 h of exposure, without inhibition. Images were captured using a digital zoom on ×20 magnification. The scale bars represent 100 µm for standard images and 50 µm for zoomed images. In all nanobioconjugates, the first three channels show nuclei labeled with Hoechst (blue), lysosomes with Lysotracker Green (green), and nanobioconjugates with Rhodamine-B (red). Yellow areas indicate colocalization between the red and green channels, suggesting lysosomal entrapment.

**FIGURE 8:**
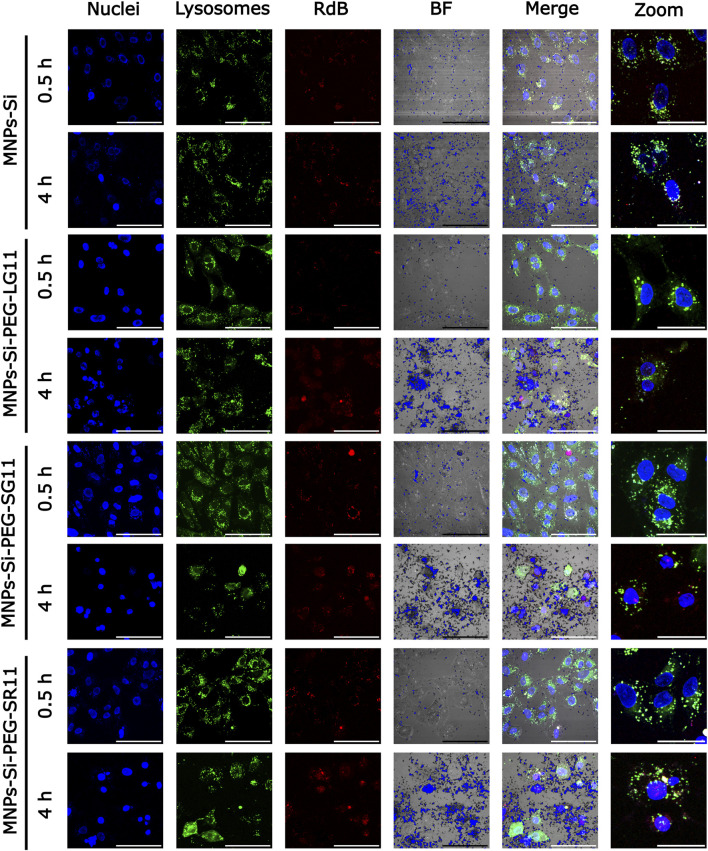
Confocal microscopy images for cell internalization and endosomal escape analysis in Vero cells at 0.5 h and 4 h of exposure, with clathrin-mediated endocytosis inhibition. Images were captured using a digital zoom on ×20 magnification. The scale bars represent 100 µm for standard images and 50 µm for zoomed images. In all nanobioconjugates, the first three channels show nuclei labeled with Hoechst (blue), lysosomes with Lysotracker Green (green), and nanobioconjugates with Rhodamine-B (red). Yellow areas indicate colocalization between the red and green channels, suggesting lysosomal entrapment.

**FIGURE 9:**
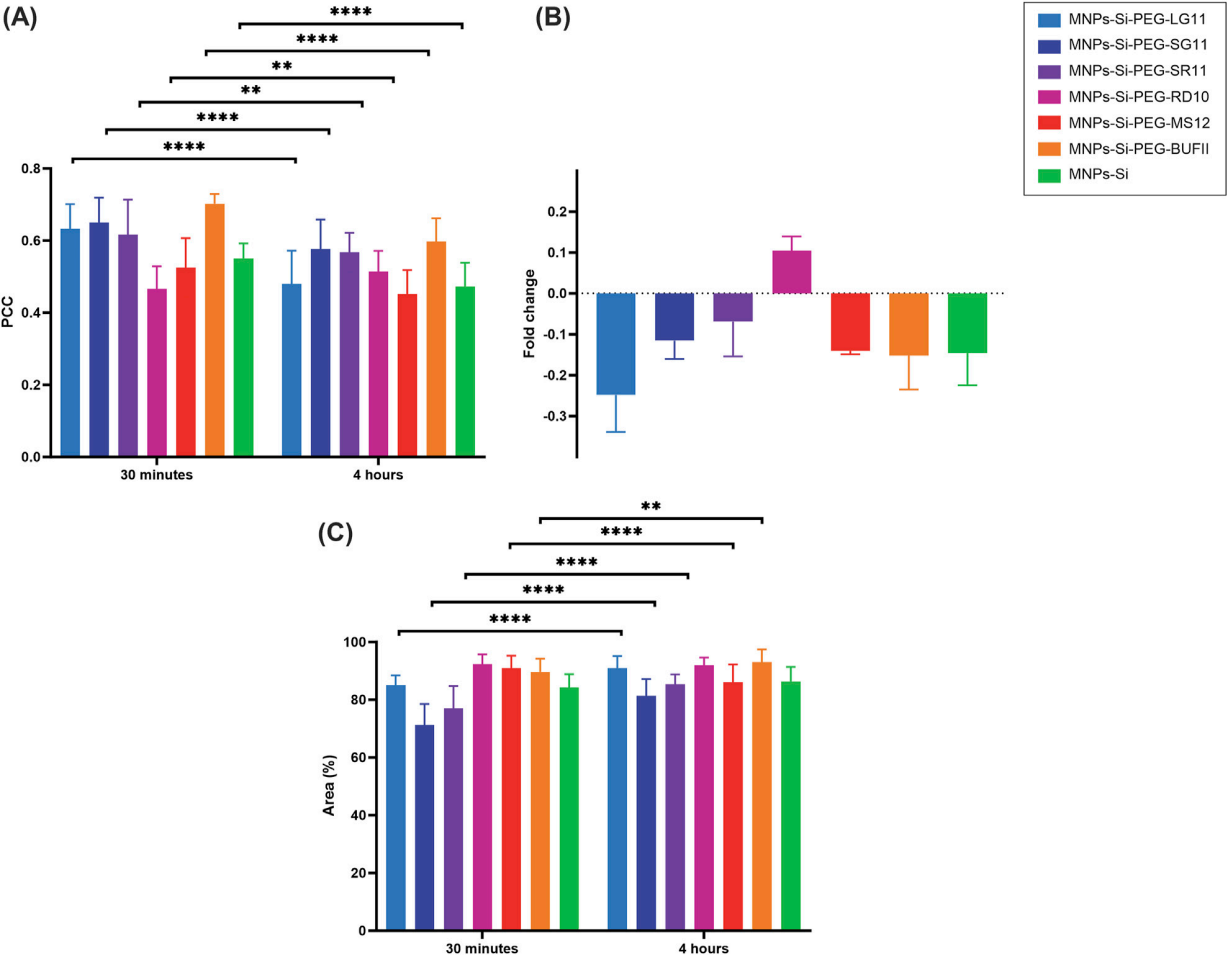
Cell-internalization and endosomal escape analysis without inhibition for the designed and comparative nanobioconjugates in Vero cells at 0.5 h and 4 h of exposure. **(A)** Pearson Correlation Coefficient (PCC), where higher values indicate stronger correlation between the red and green channels, suggesting endo/lysosomal entrapment. **(B)** Fold change analysis of the PCC. **(C)** Percentage of intracellular area covered by nanobioconjugates. Statistical significance is denoted as follows: * for p-values between 0.01 and 0.05, ** for p-values between 0.001 and 0.01, *** for p-values between 0.0001 and 0.001, and **** for p-values less than 0.0001.

**FIGURE 10:**
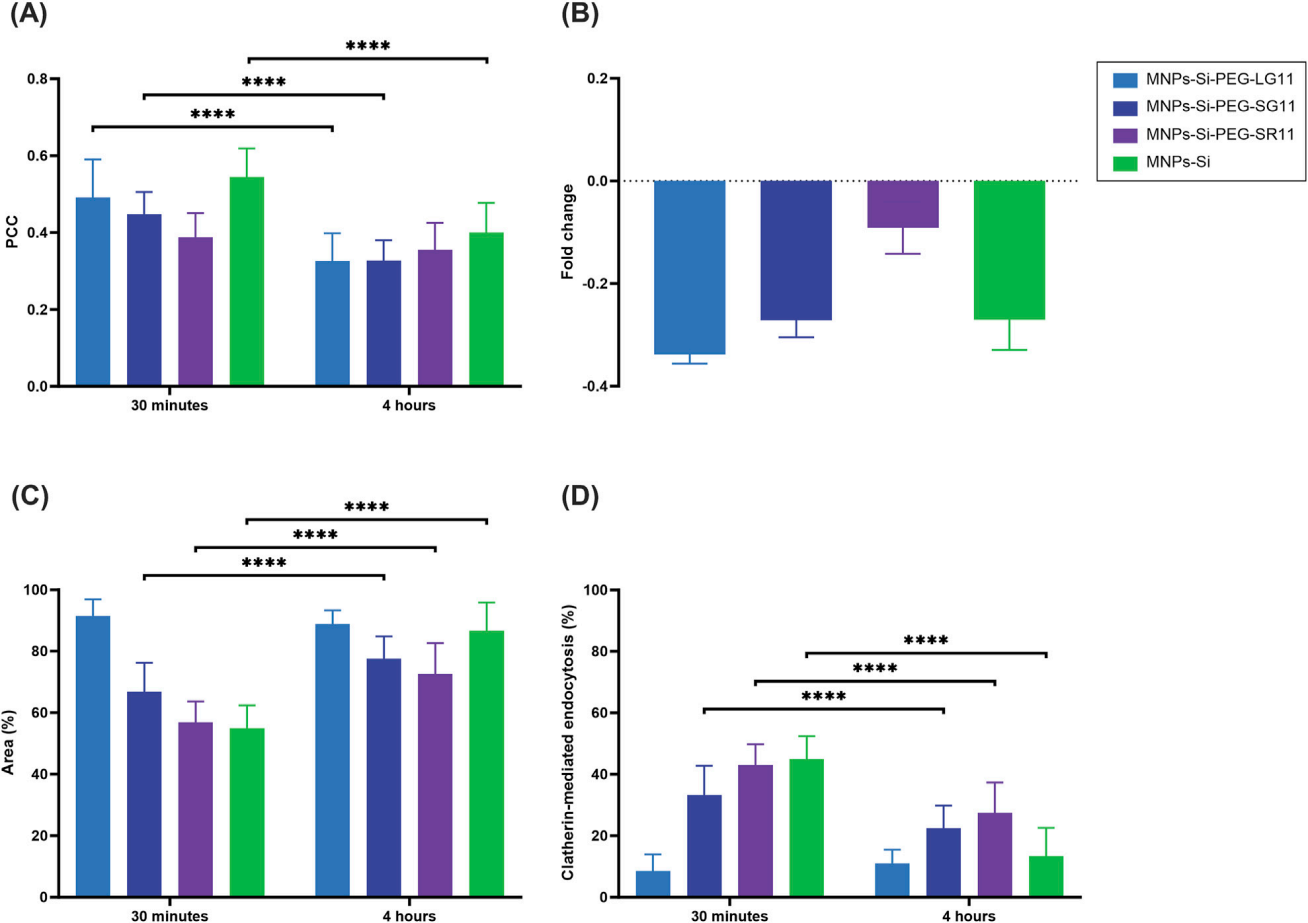
Cell-internalization and endosomal escape analysis with clathrin-mediated endocytosis inhibition of the designed nanobioconjugates in Vero cells at 0.5 h and 4 h of exposure. **(A)** Pearson Correlation Coefficient (PCC), where higher values indicate stronger correlation between the red and green channels, suggesting endo/lysosomal entrapment. **(B)** Fold change analysis of the PCC. **(C)** Percentage of intracellular area covered by nanobioconjugates. **(D)** Percentage of cellular uptake via clathrin-mediated endocytosis. The symbol * corresponds to a statistically significant difference with a p-value in the range of 0.01 ≤ p-value ≤ 0.05, ** to a statistically significant difference with a p-value in the range of 0.001 ≤ p-value <0.01, *** to p-value in the range of 0.0001 ≤ p-value ≤ 0.001 and **** to p-value < 0.0001.

Figures 7, 8 present the confocal images obtained. These images were used to determine the Pearson Correlation Coefficient (PCC) as an indicator of the nanoparticles’ endosomal escape or entrapment. Additionally, these images were utilized to determine the percentage of area covered by the nanoparticles, indicating cellular uptake, with and without inhibition, respectively. These results are depicted in Figures 9, 10. Figure 9 illustrates the results for the designed nanobioconjugates with immobilized peptides that allowed modification of the motif position and its exposure (LG11-, SG11-, and SR11-nanobioconjugate), as well as the non-modified MNPs. The performance of the designed nanobioconjugates was compared with nanobioconjugates immobilizing previously reported CPPs (RD10, MS12, and BUFII) (Park et al., 1996; Park et al., 2000; Henao et al., 2022; Ruiz Puentes et al., 2022). Detailed confocal images of the comparative nanobioconjugates are available in Supplementary Figure S7. To facilitate the analysis of endosomal escape, the fold change (Figure 9B) was calculated using the PCC graph (Figure 9A). In this graph, negative values indicate successful endosomal escape, while positive values indicate endosomal entrapment.

The results show that MNPs exhibit endosomal escape, which can be attributed to their surface charge in acidic pH environments. This environment causes the surface charge to become more positive, potentially inducing endosomal escape through electrostatic interactions (Ahmad et al., 2019). The LG11-nanobioconjugate exhibits enhanced endosomal escape compared to the unmodified MNPs and the SG11 and SR11 nanobioconjugates, indicating that the exposure of the LFVCR motif at the free N-terminal enhances the nanovehicle’s endosomal escape capabilities. Additionally, the LG11 nanobioconjugate demonstrated higher endosomal escape than nanobioconjugates with the previously reported CPPs: RD10, MS12, and BUFII. This suggests that the motif may facilitate the rupture of the endosomal layer, and the specific motif position in SG11 and SR11 peptides may interfere with the nanoparticles' escape mechanism. It is well-established that positively charged CPPs preferentially bind to negatively charged phospholipids. Notably, the intraluminal lipid bilayers of late endosomes are uniquely enriched with the negatively charged phospholipid bis (monoacylglycero) phosphate (BMP) (Kobayashi et al., 2001; Matsuo et al., 2004). Studies have shown that CPPs can induce membrane leakage in a BMP concentration-dependent manner, with greater activity at pH 5.5 (endosomal pH) than at physiological pH (Appelbaum et al., 2012). Other research supports that CPPs may undergo conformational changes due to the acidification inside the endosomes, which can destabilize the endosomal membrane bilayer (Erazo-Oliveras et al., 2012; Liang and Lam, 2012).

The fold change also indicated that the endosomal escape ability of SG11 and SR11 nanobioconjugates was lower than that of the MNPs. The differential effects of the SG11 and SR11 nanobioconjugates compared to the LG11 nanobioconjugate are important to acknowledge. The reduction in endosomal escape observed with SG11 and SR11 nanobioconjugates may be due to the distribution of their residues, which occlude the motif hypothesized to aid in endosomal escape. Comparing these results with those of the RD10 nanobioconjugate, which does not promote endosomal escape and tends to remain entrapped within endosomes to a greater extent, suggests that the absence of an exposed motif in SG11 and SR11 nanobioconjugates reduces the efficacy of endosomal escape, aligning their behavior more closely with the RD10 nanobioconjugate.

The cell covered area percentage for each conjugate is presented in Figure 9C. The results indicate that the designed nanobioconjugates showed an increase in cell uptake by Vero cells between 30 min and 4 h of exposure. At the 4-h mark, the LG11-nanobioconjugate, which has the most exposed motif, exhibited the highest internalization compared to SG11 and SR11 nanobioconjugates, which did not surpass the internalization level of the MNPs. Although the LG11-nanobioconjugate had lower internalization at 30 min, it significantly increased after 4 h, surpassing the MS12-nanobioconjugate and approaching the levels of the RD10 and BUFII nanobioconjugates. The initially lower uptake of the LG11-nanobioconjugate and the higher uptake of the comparative nanobioconjugates at 30 min can be associated with the fact that cationic nanoparticles generally exhibit higher uptake than anionic nanoparticles, due to repulsive forces between the anionic particles and the cell membrane (Karlsson et al., 1999). Cationic nanoparticles can initially utilize energy-independent mechanisms for cellular uptake (Rueda-Gensini et al., 2020), facilitated by electrostatic interactions between positively charged amino acids and negatively charged sulfonated glycoproteins, which destabilize the cell membrane (Fröhlich, 2012). However, cationic particles can also enter cells via energy-dependent pathways (Rueda-Gensini et al., 2020). On the other hand, anionic nanoparticles primarily rely on energy-dependent mechanisms such as macropinocytosis, clathrin-mediated endocytosis, and caveolin-mediated endocytosis for internalization (Harush-Frenkel et al., 2007; Martín et al., 2011; Neves-Coelho et al., 2017; Sousa de Almeida et al., 2021). The significant increase in LG11-nanobioconjugate uptake after 4 h suggests that energy-dependent pathways are slower than energy-independent mechanisms, leading to delayed but substantial uptake of the anionic LG11-nanobioconjugate. The authors hypothesized that this slower uptake might be associated with the remarkable potential of the nanobioconjugate for endosomal escape.

The inhibition of the clathrin-mediated route was assessed for the nanobioconjugates to determine the effect of motif position on cellular uptake and endosomal escape, as clathrin-mediated endocytosis represents a significant route for nanoparticle uptake (Rappoport et al., 2012; Palocci et al., 2017). As depicted in Figure 10B, the fold change of the PCC values (shown in Figure 10A) for all nanobioconjugates slightly decreased at both exposure times compared to values prior to inhibition (Figure 9B). Clathrin-mediated endocytosis is one of the main routes by which internalized nanoparticles end up in endosomes (Doherty and McMahon, 2009; McMahon and Boucrot, 2011; Hu et al., 2015), explaining the observed decrease in PCC percentages. These results are consistent with the previous findings, showing that the LG11-nanobioconjugate still exhibited the highest endosomal escape. The uptake of nanobioconjugates by Vero cells, following clathrin-mediated endocytosis inhibition, revealed also important insights (Figure 10C). The inhibition mainly affected the uptake of the SR11-nanobioconjugate and the MNPs at the 30-min mark, indicating that their initial uptake is largely mediated by clathrin-dependent endocytosis. Over time, these nanoparticles were internalized by alternative routes. Notably, the LG11-nanobioconjugate, with the exposed motif, exhibited the highest area coverage percentages, surpassing 80% at both time points studied. This suggests that the LG11-nanobioconjugate predominantly utilizes alternative endocytic pathways such as macropinocytosis, caveolin-mediated endocytosis, or clathrin/caveolin-independent endocytosis for cellular uptake.

Figure 10D provides a clearer depiction of the percentage of cellular uptake by internalization mechanisms for each nanobioconjugate. This value was estimated from the cell area coverage percentages previously obtained. The results reveal that clathrin-mediated internalization occurs at a higher rate within the first 30 min for almost all nanobioconjugates compared to a longer exposure period of 4 h. Notably, the LG11-nanobioconjugate displays a distinct pattern. It not only has the lowest percentage of uptake via this route but also shows a slight increase from 8.57% at 30 min to 11.1% at 4 h. Despite this lower entry percentage, the LG11-nanobioconjugate demonstrates the highest overall uptake, indicating that it relies more heavily on alternative endocytic pathways for cellular uptake. As previously noted, well-known routes for the transportation of IONPs include caveolin-mediated endocytosis and macropinocytosis (Martín et al., 2011; Neves-Coelho et al., 2017; Salloum et al., 2019; Rueda-Gensini et al., 2020). The uptake of the LG11-nanobioconjugate might be primarily influenced by its surface charge over long-term uptake, as its behavior is similar to that of the anionic bare MNPs. However, the initial low uptake via the clathrin-mediated route is not likely due to its surface charge, as the bare MNPs showed higher uptake. This suggests that the motif influences the activation of other internalization mechanisms. To fully understand the internalization mechanisms of the LG11-nanobioconjugate and the impact of its exposed motif, it is crucial to investigate alternative routes such as caveolin-mediated endocytosis, clathrin- and caveolin-independent endocytosis, and phagocytosis (Doherty and McMahon, 2009; McMahon and Boucrot, 2011; Bohdanowicz and Grinstein, 2013; Qiu et al., 2023). For the SR11 and SG11 nanobioconjugates, where the motif is not exposed, cellular uptake may be mediated by their cationic charge, potentially through energy-independent pathways like direct penetration (Szabó et al., 2021). Moreover, previous studies have demonstrated that cationic MNPs can induce endocytosis via alternative pathways such as micropinocytosis (Pang et al., 2015; Rueda-Gensini et al., 2020; Li and Pang, 2021; Szabó et al., 2021).

Overall, these results have demonstrated that the position of the motif within the peptides significantly affects cellular uptake. The three peptides evaluated, all containing the motif of interest, exhibited varying internalization percentages through the clathrin-mediated pathway. This observation indicates that both the motif itself and its position within the peptide structure influence the internalization mechanisms. For instance, the LG11-nanobioconjugate showed the least internalization through the clathrin-mediated route, suggesting that its exposed motif predominantly influences the peptide’s alternative uptake mechanisms.

## Conclusion

In this study, we evaluated the effect of the position of the cell-penetrating motif (LFVCR) within peptide-based magnetite nanobioconjugates on biocompatibility, cellular uptake, endosomal escape, and clathrin-mediated endocytosis activation. All peptide sequences displayed the same physicochemical characteristics in terms of size, molecular weight, net charge at pH 7, GRAVY, pI, hydrophobicity, hydrophobic moment, and Boman Index. However, slight differences were observed in the biocompatibility of the designed nanobioconjugates. Notably, the nanobioconjugate with the most exposed motif exhibited higher cellular uptake and enhanced endosomal escape compared to the other designed nanobioconjugates and those with previously reported CPPs. While clathrin-mediated endocytosis is a primary route for nanoparticle uptake, the results showed that the nanobioconjugate with the exposed motif did not primarily enter cells via this pathway, unlike the nanobioconjugates with less exposed motif. This suggests that the position of the motif significantly influences the internalization mechanisms. These results open the opportunity to study other cellular uptake routes involved in the internalization of these IONPs. The final nanobioconjugate displayed suitable characteristics in terms of size, morphology, thermal stability, biocompatibility, cellular uptake, and endosomal escape, making it promising for applications such as diagnostics, magnetic drug and gene delivery, hyperthermia, magnetic resonance imaging, and theranostics. We hope this research encourages future studies to select immobilization techniques that ensure the correct immobilization of peptides. This study highlights that proper immobilization is crucial for the functionality of the peptides, as inefficient interactions can compromise their efficacy.

## Data Availability

The original contributions presented in the study are included in the article/Supplementary Material, further inquiries can be directed to the corresponding authors.
