# Supplementary figures and images for: Evaluating the impact of cell-penetrating motif position on the cellular uptake of magnetite nanoparticles
Source: Front Bioeng Biotechnol. 2024 Dec 2;12:1450694. doi: 10.3389/fbioe.2024.1450694 (PMC11646778; doi:10.3389/fbioe.2024.1450694)

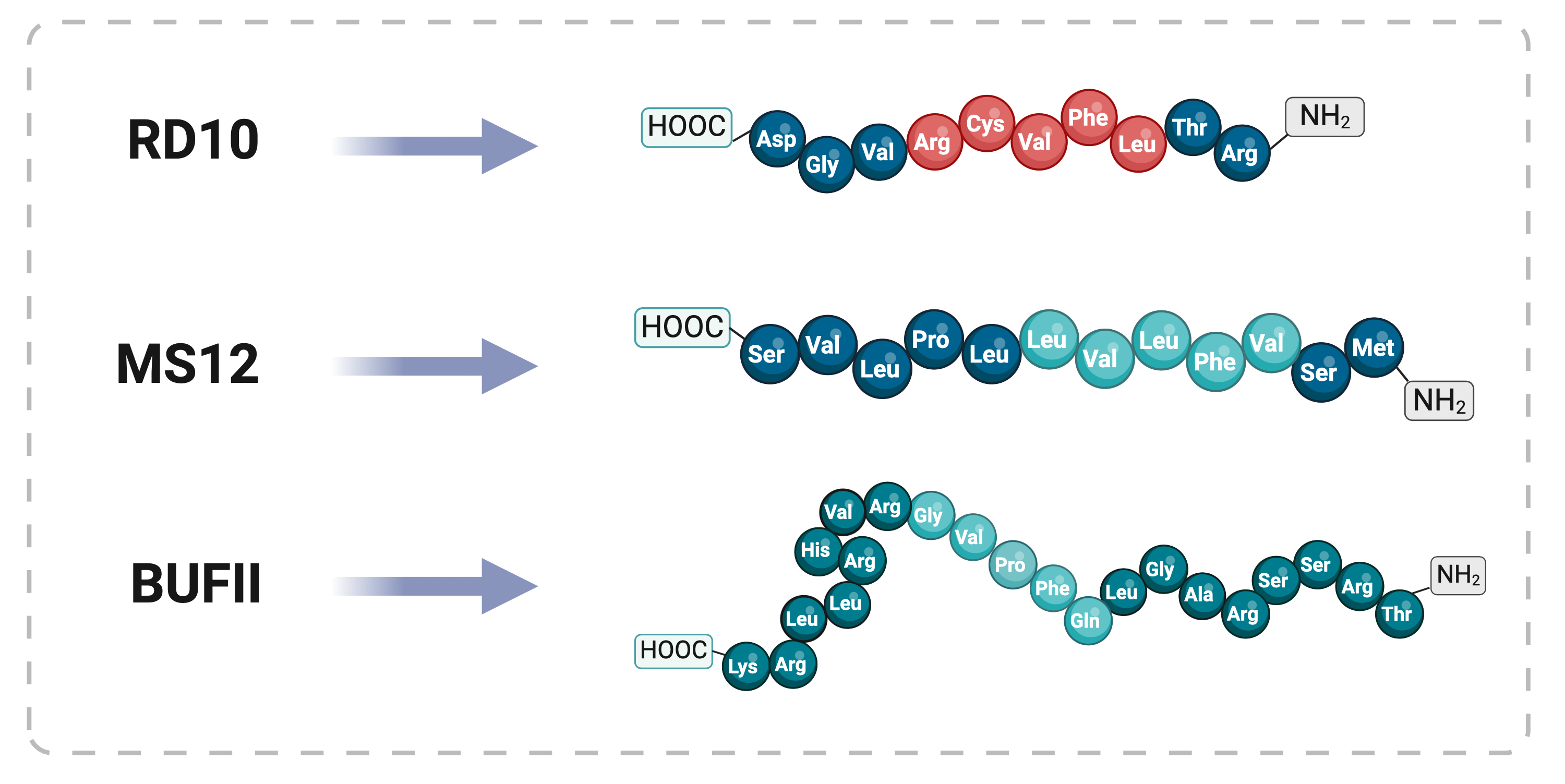

Supplement: Supplementary file 1 [file Image1.TIFF]

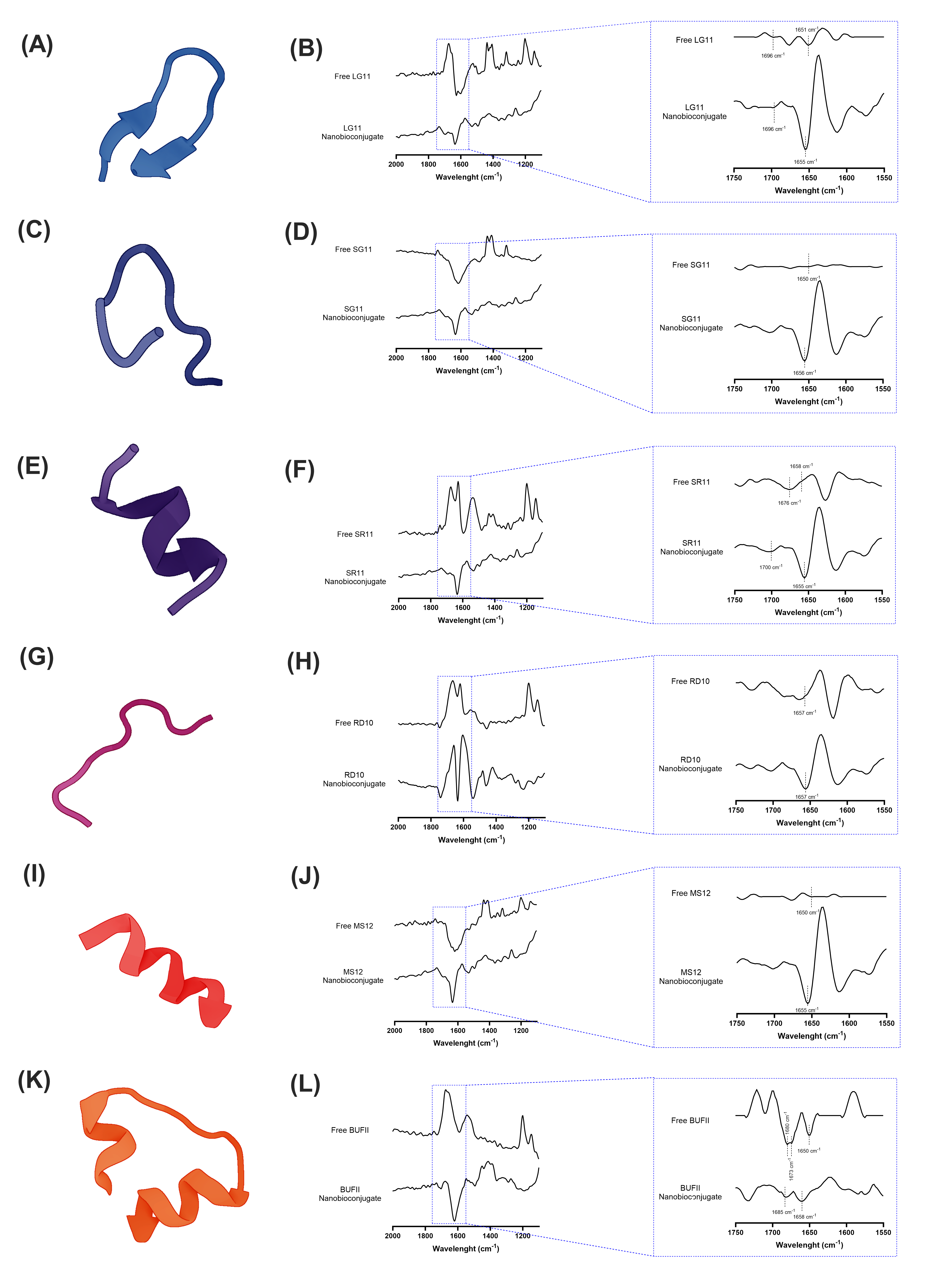

Supplement: Supplementary file 3 [file Image3.TIF]

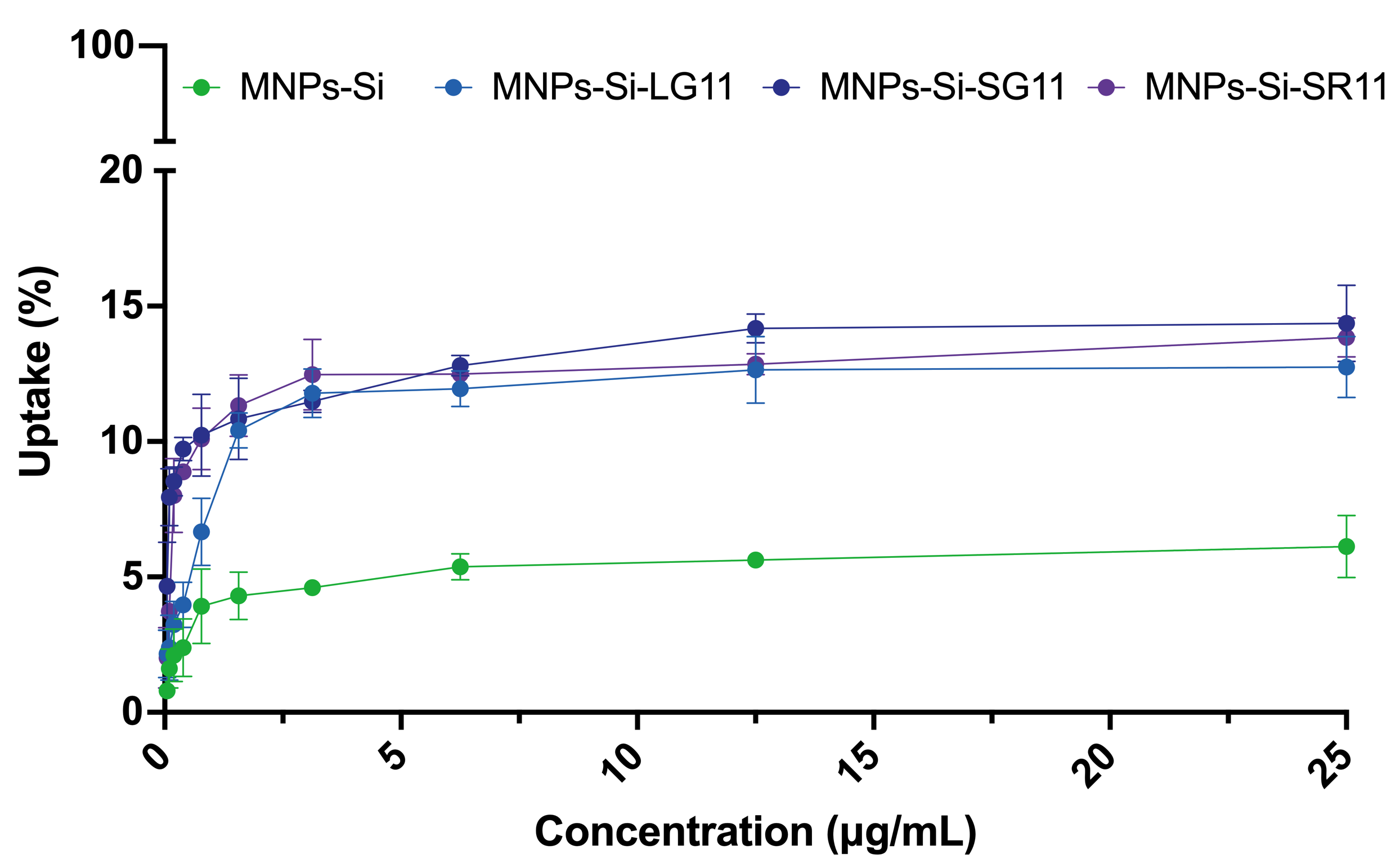

Supplement: Supplementary file 4 [file Image5.TIFF]

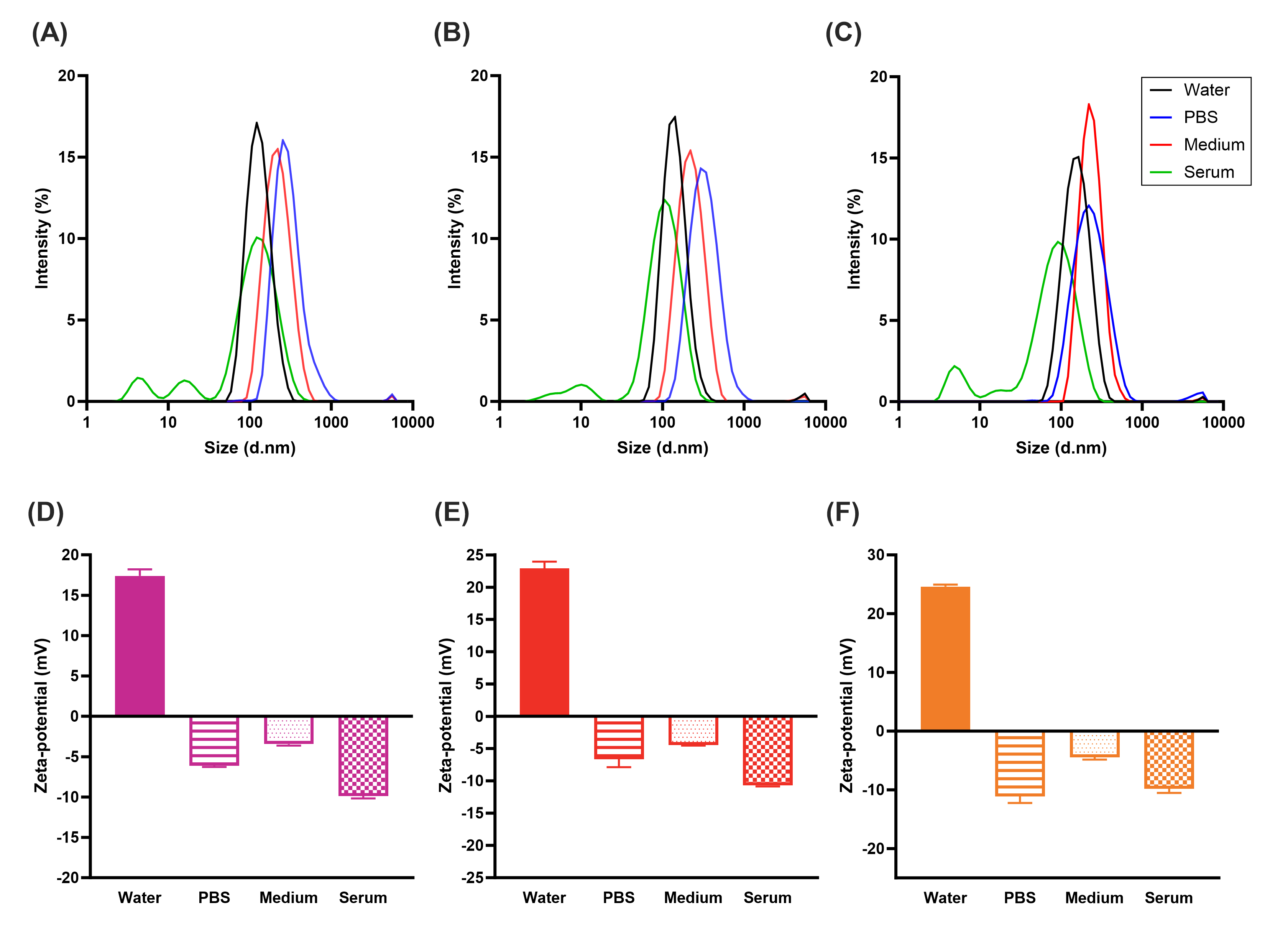

Supplement: Supplementary file 5 [file Image4.TIF]

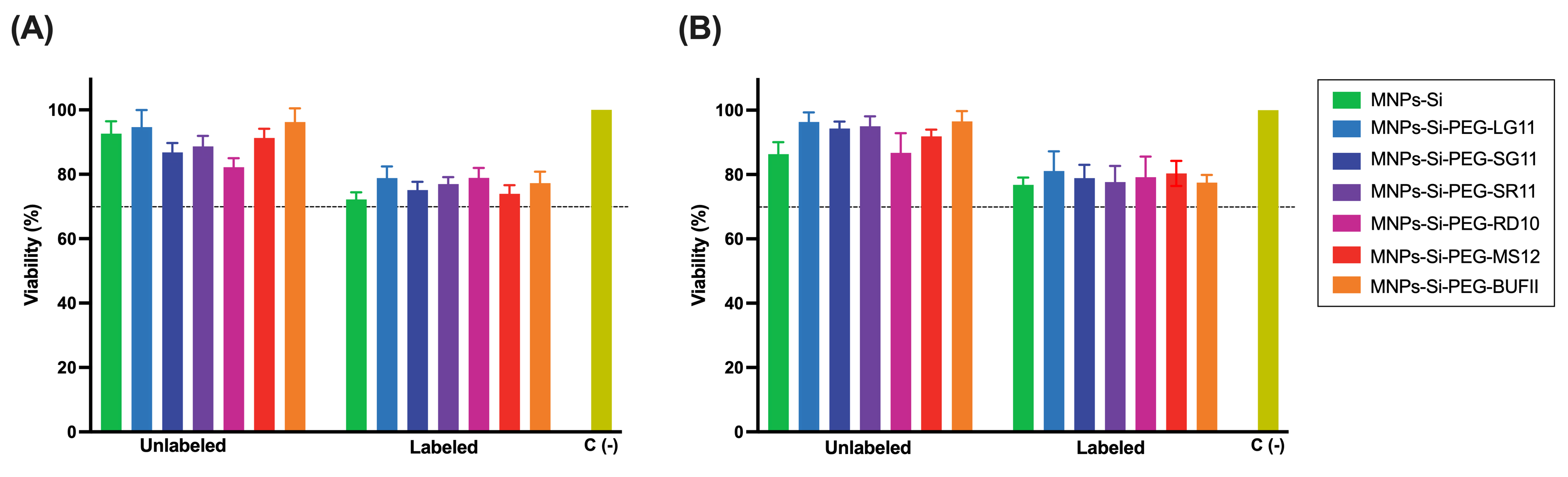

Supplement: Supplementary file 6 [file Image6.TIFF]

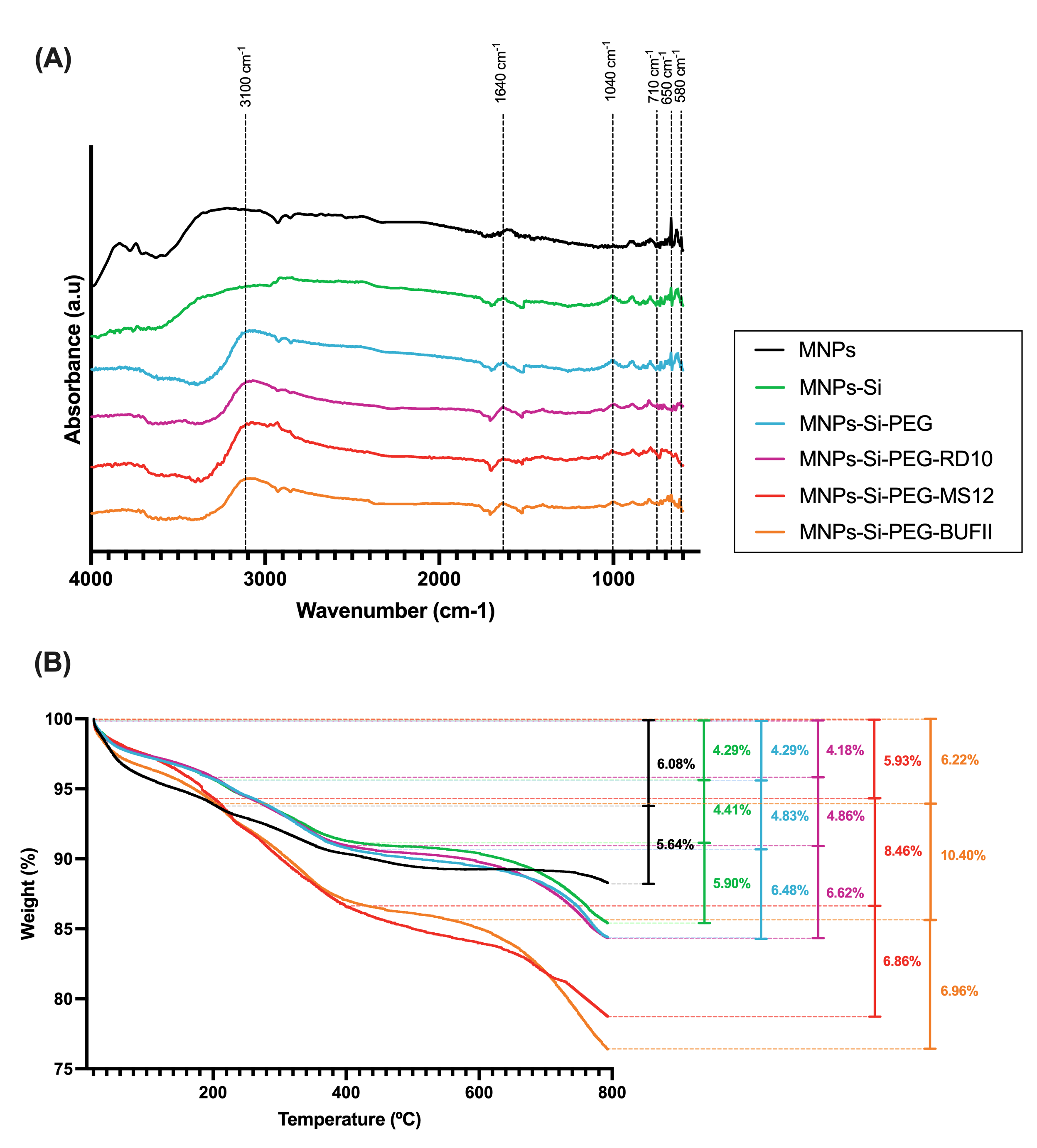

Supplement: Supplementary file 7 [file Image2.TIFF]

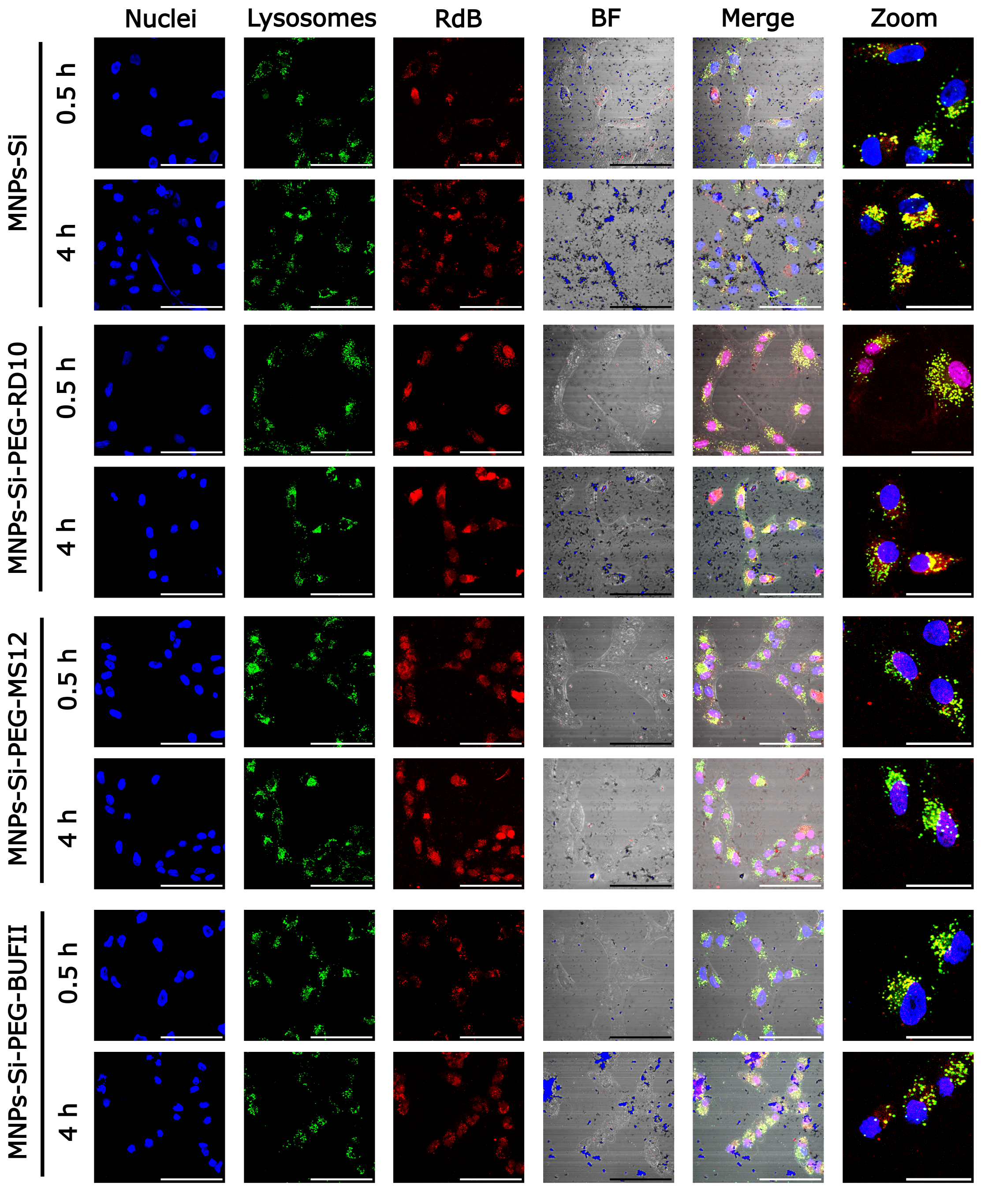

Supplement: Supplementary file 8 [file Image7.TIFF]
