# Supplementary material for: Evaluating the impact of cell-penetrating motif position on the cellular uptake of magnetite nanoparticles
Source: Front Bioeng Biotechnol. 2024 Dec 2;12:1450694. doi: 10.3389/fbioe.2024.1450694 (PMC11646778; doi:10.3389/fbioe.2024.1450694)
Supplement: Supplementary file 2 [file Table1.DOCX]

Supplementary Material

**Supplemental Methods**

**Platelets uptake**

The uptake of nanobioconjugates was determined by measuring fluorescence changes before and after membrane lysis induced by Triton X-100. Starting from an initial concentration of 25 µg/mL, an encapsulation curve was obtained by serially diluting the Rhodamine B-labeled nanobioconjugates in PBS 1X using a 96-well microplate. Platelet-rich plasma (PRP) was obtained as described in the platelet aggregation assay, and a 1:100 dilution was prepared using PBS 1X (2500 platelets/µL). The nanobioconjugate dilutions were mixed with 100 µL of the PRP solution at a 1:1 volume ratio. After a 30-minute incubation at 37°C and 110 rpm, the initial fluorescence intensity was recorded. Subsequently, 10 µL of Triton X-100 was added to each well to induce membrane lysis. After an additional 10-minute incubation at 37°C and 110 rpm, fluorescence intensity was measured again. The increase in fluorescence intensity following membrane lysis was interpreted as the release of encapsulated nanobioconjugates. Fluorescence measurements were taken using a spectrofluorometer (FluoroMax Plus C, Horiba, Japan) with excitation and emission wavelengths set at 546 nm and 568 nm, respectively. The uptake percentage was calculated using Equation (4).

| $\boldsymbol{Uptake}\left( \boldsymbol{\%} \right)\boldsymbol{=}\left[ \frac{\boldsymbol{FI}_{\boldsymbol{after}}\boldsymbol{-}\boldsymbol{FI}_{\boldsymbol{before}}}{\boldsymbol{FI}_{\boldsymbol{after}}} \right]\boldsymbol{*100\%}$ | (4) |
| --- | --- |

Here, ${FI}_{before}$ is the fluorescence intensity emission before the addition of Triton X-100, and ${FI}_{after}$ is the fluorescence intensity emission after the membrane lysis with the addition of Triton X-100.

**Supplemental Tables and Figures**

**Supplemental Table 1.** Efficiency of peptide immobilization on PEGylated MNPs.

| **Nanobioconjugate** | **Immobilization efficiency** |
| --- | --- |
| MNPs-Si-PEG-LG11 | 87.0% |
| MNPs-Si-PEG-SG11 | 86.0% |
| MNPs-Si-PEG-SR11 | 85.0% |
| MNPs-Si-PEG-RD10 | 57.0% |
| MNPs-Si-PEG-MS12 | 80.8% |
| MNPs-Si-PEG-BUFII | 91.0% |

**Supplemental Table 2.** DLS and zeta potential test results for the comparative nanobioconjugates, showing nanoparticle size, polydispersity index, and surface charge in water at pH 7.4 (water), PBS 1X (PBS), DMEM (medium), and DMEM + 10% (v/v) FBS (serum).

| **Solution** | **Nanobioconjugate** | **Average diameter (nm)** | **Polydispersity index** | **Zeta-potential (mV)** |
| --- | --- | --- | --- | --- |
| Water | MNPs-Si-PEG-RD10 | 135.8 ± 2.902 | 0.238 ± 0.015 | 35.19 ± 0.704 |
|  | MNPs-Si-PEG-MS12 | 128.9 ± 1.514 | 0.205 ± 0.017 | 34.86 ± 0.379 |
|  | MNPs-Si-PEG-BUFII | 159.5 ± 4.661 | 0.246 ± 0.025 | 24.60 ± 0.351 |
| PBS | MNPs-Si-PEG-RD10 | 294.5 ± 35.55 | 0.208 ± 0.039 | −6.12 ± 0.145 |
|  | MNPs-Si-PEG-MS12 | 304.9 ± 28.03 | 0.151 ± 0.001 | −6.66 ± 1.20 |
|  | MNPs-Si-PEG-BUFII | 213.9 ± 4.065 | 0.210 ± 0.010 | −11.1 ± 1.15 |
|  | MNPs-Si-PEG-RD10 | 212.4 ± 3.889 | 0.171 ± 0.040 | −3.44 ± 0.200 |
| Medium | MNPs-Si-PEG-MS12 | 208.9 ± 2.101 | 0.130 ± 0.046 | −4.47 ± 0.0802 |
|  | MNPs-Si-PEG-BUFII | 240.3 ± 12.24 | 0.195 ± 0.025 | −4.48 ± 0.365 |
| Serum | MNPs-Si-PEG-RD10 | 142.5 ± 4.219^α^ | 0.629 ± 0.054 | −9.91 ± 0.653 |
|  |  | 12.10 ± 6.393^β^ |  |  |
|  | MNPs-Si-PEG-MS12 | 116.1 ± 2.113^α^ | 0.409 ± 0.071 | −10.7 ± 0.153 |
|  |  | 9.335 ± 2.830^β^ |  |  |
|  | MNPs-Si-PEG-BUFII | 98.64 ± 1.389^α^ | 0.628 ± 0.001 | −9.81 ± 0.711 |
|  |  | 5.417 ± 0.5803^β^ |  |  |

^α^Refers to the first (maximum) peak.

^β^Refers to the second peak.

**Supplemental Figure 1.** General structure of the comparative peptides RD10, MS12, and BUFII. The amino acids are represented by three-letter codes, with the motif sequence highlighted in red and shown with its respective chemical structure. Created with BioRender.com.

**Supplemental Figure 2.** Physicochemical characterization of the comparative nanobioconjugates immobilized with RD-10, MS-12 and BUF-II peptides**. (A)** FTIR spectra, and **(B)** TGA analysis.

**Supplemental Figure 3.** Graphical representation of the peptide secondary structures for **(A)** LG11, **(C)** SG11, **(E)** SR11, **(G)** RD10, **(I)** MS12, and **(K)** BUFII, generated using PEP-FOLD3. FTIR spectra and second derivative analyses for **(B)** LG11, **(D)** SG11, **(F)** SR11, **(H)** RD10, **(J)** MS12, and **(L)** BUFII nanobioconjugates, along with their respective free peptides.

**Supplemental Figure 4.** DLS intensity plots of comparative nanobioconjugates dispersed in water at pH 7.4 (water), PBS 1X (PBS), DMEM (medium), and DMEM + 10% (v/v) FBS (serum) for **(A)** RD10, **(B)** MS12, and **(C)** BUFII nanobioconjugates. Zeta potential measurements of the nanobioconjugates under the same conditions for **(D)** RD10, **(E)** MS12, and **(F)** BUFII.

**Supplemental Figure 5.** Percentage of platelet uptake for bare MNPs and nanobioconjugates.

**Supplemental Figure 6.** Cell viability of Vero cells exposed to unlabeled and Rhodamine B-labeled nanobioconjugates for **(A)** 0.5 hours and **(B)** 4 hours. Non-treated cells were employed as negative control.

**Supplemental Figure 7.** Confocal microscopy images for cell internalization and endosomal escape analysis in Vero cells after 0.5 h and 4 h of exposure to the comparative nanobioconjugates, without inhibition. Images were captured using digital zoom on 20X magnification. The scale bars represent 100 µm for standard images and 50 µm for zoomed images. In all nanobioconjugates, the channels display nuclei labeled with Hoechst (blue), lysosomes with Lysotracker Green (green), and nanobioconjugates with Rhodamine-B (red). Yellow areas indicate colocalization between the red and green channels, suggesting lysosomal entrapment.
